# Food knowledge depends upon the integrity of both sensory and functional properties: a VBM, TBSS and DTI tractography study

**DOI:** 10.1038/s41598-019-43919-8

**Published:** 2019-05-15

**Authors:** Miriam Vignando, Marilena Aiello, Adriana Rinaldi, Tatiana Cattarruzza, Giulia Mazzon, Paolo Manganotti, Roberto Eleopra, Raffaella I. Rumiati

**Affiliations:** 1 Area of Neuroscience, SISSA, Trieste, Italy; 2 Department of Neurology, “S. Maria della Misericordia” University Hospital, Udine, Italy; 3 Department of Medical, Surgical and Health Sciences, Clinical Neurology Unit, Cattinara University Hospital, Trieste, Italy; 4 ANVUR, Rome, Italy

**Keywords:** Language, Dementia, Alzheimer's disease

## Abstract

Food constitutes a fuel of life for human beings. It is therefore of chief importance that their recognition system readily identifies the most relevant properties of food by drawing on semantic memory. One of the most relevant properties to be considered is the level of processing impressed by humans on food. We hypothesized that recognition of raw food capitalizes on sensory properties and that of transformed food on functional properties, consistently with the hypothesis of a sensory-functional organization of semantic knowledge. To test this hypothesis, patients with Alzheimer’s disease, frontotemporal dementia, primary progressive aphasia, and healthy controls performed lexical-semantic tasks with food (raw and transformed) and non-food (living and nonliving) stimuli. Correlations between task performance and local grey matter concentration (VBM) and white matter fractional anisotropy (TBSS) led to two main findings. First, recognition of raw food and living things implicated occipital cortices, typically involved in processing sensory information and, second, recognition of processed food and nonliving things implicated the middle temporal gyrus and surrounding white matter tracts, regions that have been associated with functional properties. In conclusion, the present study confirms and extends the hypothesis of a sensory and a functional organization of semantic knowledge.

## Introduction

Semantic memory stores the information about all we know, including sensory and abstract properties of objects. Over the years, scholars have focused on defining the organizing principles of semantic knowledge in the brain, and proposed different models of semantic memory. The neuropsychological observations of patients with a disproportionate impairment at recognizing items from a particular category or domain of knowledge while sparing others imposed a great impulse on the field. For instance, the double dissociation in recognizing living things and nonliving things has been taken by Warrington, Shallice, and colleagues as evidence that semantic knowledge is likely to be organized in subsystems, coding sensory properties and functional properties respectively^1,2^. According to the sensory-functional hypothesis (SFH), damage to the sensory subsystem would impair recognition of living things, on the ground that for recognizing these items, the integrity of their sensory properties such as the colour, the shape, the texture is necessary; likewise, damage to the functional subsystem would impair recognition of nonliving things, because for recognizing these items their functional properties (how to manipulate them, what they can be used for) need to be preserved.

However, patients’ performance might breakdown in a finer grain. This is the case, for instance, of patient E.W. who had a selective deficit at naming animals but could still recognize other living categories^3^. To accommodate for such cases, Caramazza and collaborators suggested that the organization of our knowledge in the brain has been shaped by evolutionary pressure (Distributed Domain Specific Hypothesis, DDSH)^4^, allowing a faster recognition of items that are more relevant for our survival: conspecifics, animals, plants and, perhaps, tools. In their view, all the properties of the items belonging to a specific category are stored together in the brain; therefore, brain damage might end up affecting more modalities, irrespective of whether the items are living or nonliving.

However, recent imaging and computational studies provided further evidence in support of the anatomical segregation of living things and non-living things: (see e.g.^5–9^): recognition of the living things tends to occur in visual regions and that of utensils in the posterior parietal regions, while semantic knowledge depends upon the integrity of the temporal lobe (see the ‘spoke-and-hub’ model^5–9^).

In the present study, we exploit food as a stimulus to test these different hypotheses about object recognition (see^10^). In particular, we focused on the level of food transformation whereby natural, unprocessed food, such as apples or lettuce, can be distinguished from transformed food that has been manufactured, such as pizza or a hamburger. The importance of food transformation has been extensively addressed by Richard Wrangham^11^. Specifically, he argued that food transformation procedures are ubiquitous and have contributed toward the evolution of our species by providing a greater net energetic gain. Moreover, cooking food reduces the risk of ingesting poisonous substances and makes it more palatable. The relevance of the level of transformation in food recognition has recently been supported by two electrophysiological studies with healthy participants. First, evidence that the human brain can discriminate whether a food is natural or transformed at around 120 milliseconds has recently been provided (see Coricelli *et al*., under review). Second, the N400, that is a measure of semantic incongruency, was found to be greater for trials pairing transformed food images with sentences describing sensory properties, and natural food images with sentences describing functional properties^12^. These results support the hypothesis, recently put forward and extending the SFH^10^, that recognition of natural food and living things is alike, as it relies more efficiently on sensory properties, whereas recognition of transformed foods relies more efficiently on functional properties, as it happens for recognition of artefacts. The aim of our study was therefore to test this hypothesis. Specifically, we expected knowledge about living things and natural food to be represented in the lateral occipital cortex (LO) and the fusiform gyrus (FG), as well as in sensory processing areas (e.g.^13^), and knowledge about nonliving things and transformed food, in regions such as the inferior frontal gyrus (IFG), left posterior middle temporal gyrus (pMTG), and superior temporal gyrus (STG), previously associated to knowledge of tools (e.g.^13–15^). This organization should also be mirrored in the involvement of white matter tracts. If these hypotheses were confirmed, our findings would strongly support the view that semantic memory is organized, at least partially, in sensory/functional subsystems.

In order to test these hypotheses, in the present study we assessed the semantic knowledge about food (natural and transformed) and non-food (living and nonliving things) in healthy participants and in patients with primary progressive aphasia (PPA), Alzheimer’s disease and behavioural frontotemporal degeneration (bvFTD). All these different neurodegenerative disorders are characterized by extensive damage to the temporal lobe that has reliably been associated with semantic memory^16^. Participants’ accuracy on tests tapping semantic knowledge about food and non-food items was correlated with the local grey matter concentration and the water diffusion of the white matter tracts (fractional anisotropy, FA).

## Results

### Behavioral analyses

Behavioral analyses are reported in the Supplemental material S3–S6.

### Anatomical analyses

#### VBM and TBSS

**Figure 1:**
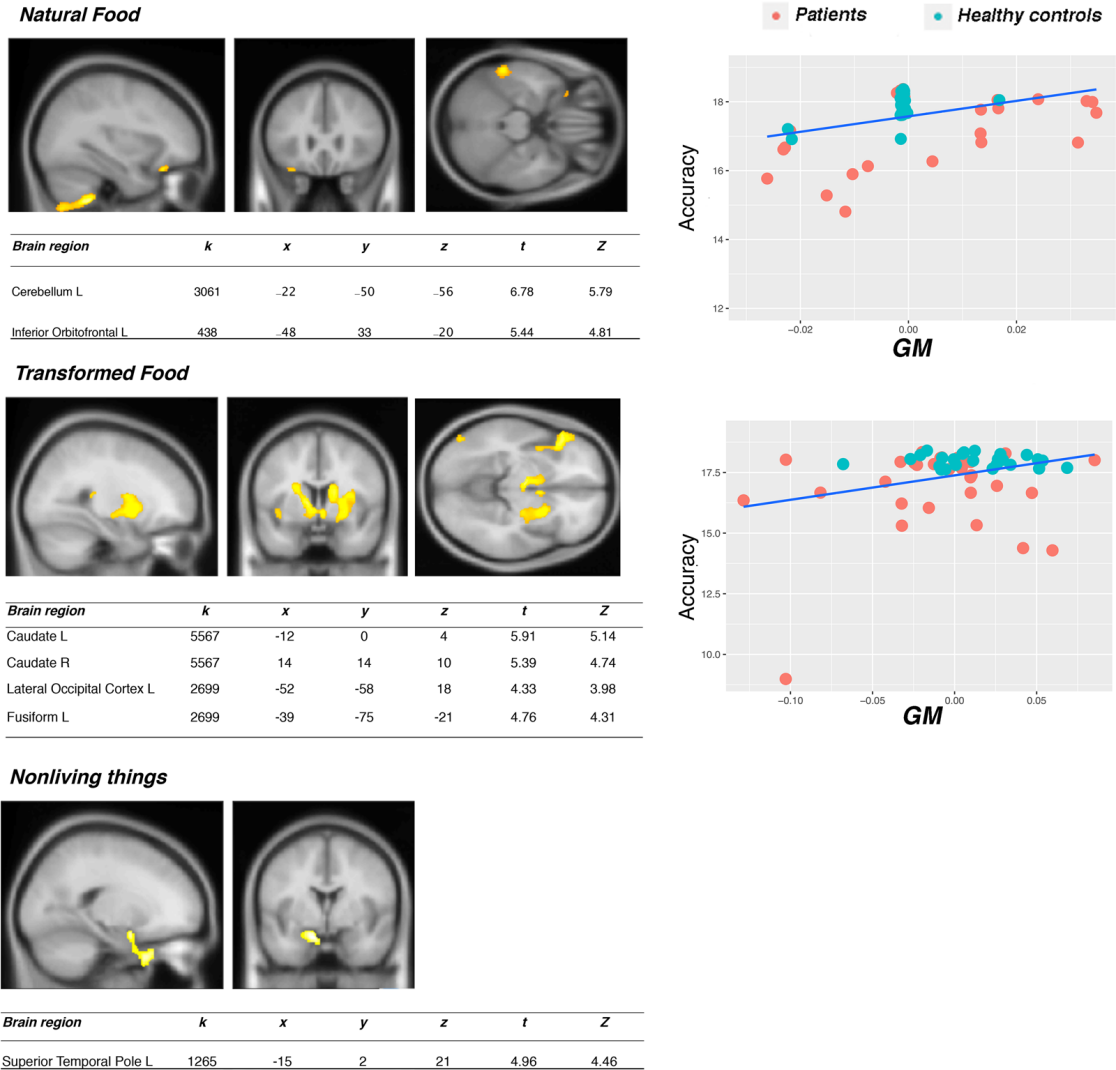
Significant association between recognition of natural food, transformed food, living things and non-living things in the word-picture matching task and gray matter volume (p FWE < 0.05). K = cluster size (number of voxels). The insets show the scatter plots obtained by correlating the GM volume for each of our participants’ scans at that cluster with the behavioural variable of interest. On the y axis accuracy is reported, on the x axis GM values are reported.

Association between gray matter and recognition of natural food, transformed food, living things, nonliving things: The tests (naming, word-picture matching, categorization) we devised aimed at tapping the participants’ semantic knowledge about natural food, transformed food, living things, and non-living things. Thus, by correlating the participants’ performance on the different tasks for each semantic category with voxel-wise GM density, we can identify the neural correlates underlying the above four semantic categories of interest. In the ***Naming task***, only the correlation between the accuracy scores for nonliving things and GM density in the posterior parietal cortex (cuneus, precuneus) was significant (See Fig. S7a). In the ***Word-Picture matching task*** (see Fig. 1, the accuracy on the natural food significantly correlated with two clusters, one in the left cerebellum, extending to the left inferior temporal cortex and to the right cerebellum, and one in the left OFC, whereas recognition of transformed food significantly correlated with a cluster peaking in the left FG, extending to the angular gyrus (AG) and posterior middle temporal gyrus (pMTG), and with a large cluster comprising the caudate nucleus (bilaterally), the left OFC and insular cortex. Recognition of nonliving things correlated with the left anterior STG (*p* uncorrected < 0.001; however, the significance did not survive multiple comparisons *p*FWE = 0.1, but we report the result in virtue of its consistency with the literature on knowledge about utensils). In the ***Categorization task***, no significant correlations survived the corrections when we considered both gray and white matter. No correlation survived corrections for the TBSS analyses for any of the categories considered.

#### Conjunction analyses in VBM and TBSS

With this analysis, we aimed at testing the hypothesis that recognition of natural food and living things relies on sensory features (“sensory knowledge”), and recognition of transformed food and nonliving things counts on functional features (“functional knowledge”). This was achieved by testing whether natural food and living things, on the one hand, and transformed food and nonliving things, on the other, share common neural substrates by exploring whether these categories have overlapping voxels. The “sensory subsystem” will, therefore, correspond to the regions shared by natural food and living things, and the “functional subsystem” will correspond to the regions shared by transformed food and non-living things. As in the previous section, we ran separate models for each task.

In the ***Naming task***, the TBSS conjunction analysis returned significant results. Naming performance correlated with the right arcuate segment (*p*FWE < 0.001) on “sensory knowledge” trials (natural food plus living things), and with the right cingulum in the temporal region (*p*FWE = 0.01) on “functional knowledge” trials (transformed food plus non-living things) (see Fig. S7b).

In the ***Word-picture matching task***, performance on “functional knowledge” trials only led to significant results. The VBM showed a significant large bilateral cluster with its peak in the left pMTG and comprising the STG, a cluster peaking in the left pallidum and extending to the posterior cingulate gyrus, and a cluster with its peak in the right rolandic operculum (IFG). The TBSS and the DTI returned a correlation with FA in the bilateral corticospinal tract, right cingulum (anterior portion) and left inferior frontal longitudinal fasciculus, as observed in the naming task (*p*FWE < 0.05).

**Figure 2:**
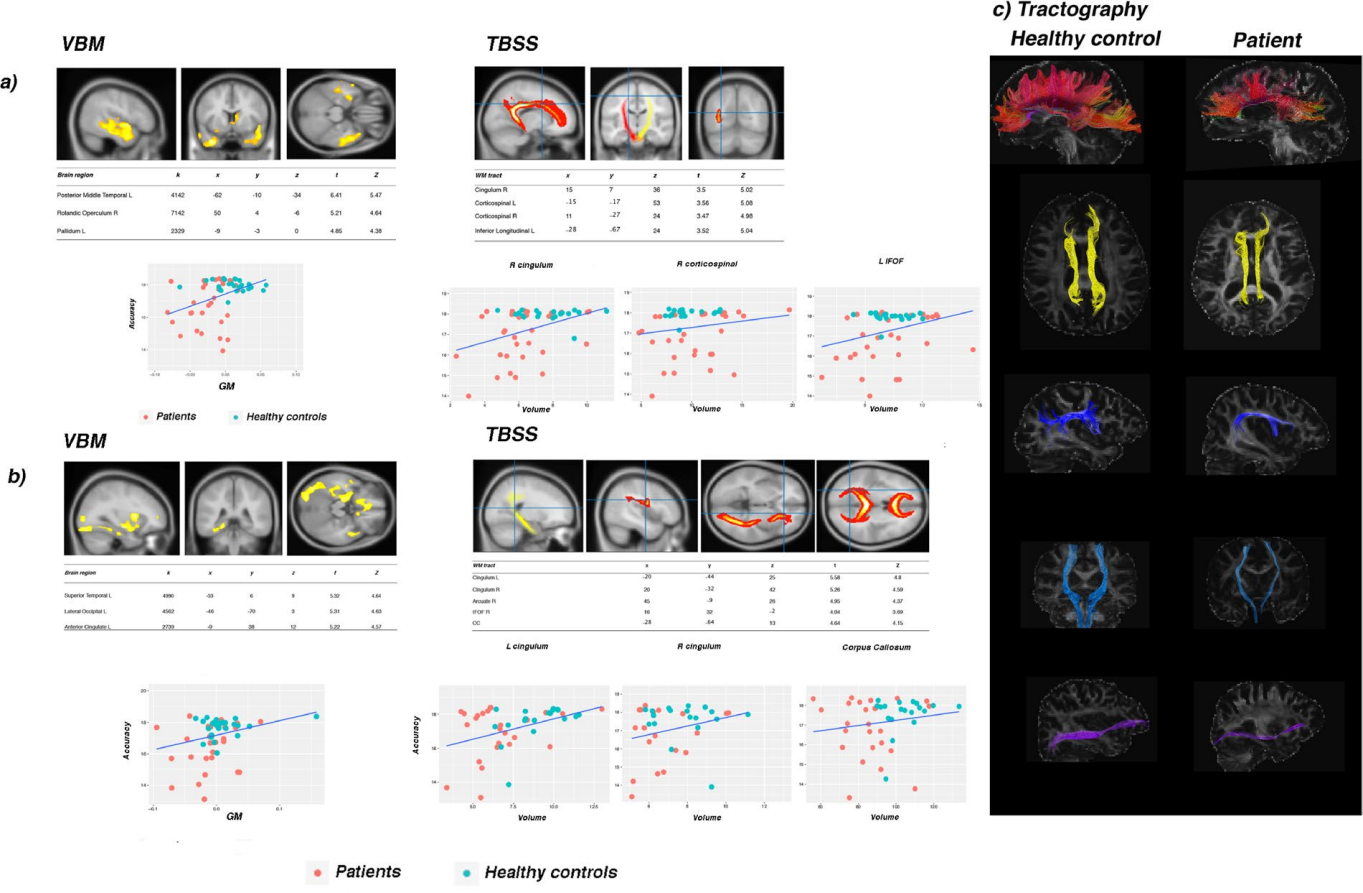
VBM and TBSS results concerning the functional and sensory knowledge, in the (**a**) word-picture matching and (**b**) categorization tasks, respectively, as described in the text. For the TBSS, the crosshair represents the coordinates corresponding to the peak cluster returned by the analysis. Images have been reconstructed as reported in the Methods section. All results reported survived the FWE correction (p FWE < 0.05). K = cluster size (number of voxels). The insets show scatter plots obtained by correlated the GM volume estimate for each of our participants’ scans at that cluster, or the volume of the white matter tracts, with the behavioural variable of interest. On the y axis accuracy is reported, on the x axis GM values or tracts’ volumes are reported. (**c**) DTI tractography. Examples of white matter tracts emerged with the TBSS analyses and hand mapped with DTI tractography. On the left, tracts of an healthy control; on the right, the same tracts are reported for a patient.

In the ***Categorization task***, performance on “sensory knowledge” trials correlated (*p*FWE < 0.05) with GM density in three main clusters: the first with its peak in the left LOC and comprising the FG; the second, bilateral, includes the anterior cingulate cortex and extends to the OFC, and a third cluster has a peak in the left superior temporal cortex extending, bilaterally, to the inferior temporal cortex, temporal pole (see Fig. 2). No other significant correlations emerged from this analysis. The TBSS conjunction analysis confirmed the VBM findings on this task, as far as the “sensory knowledge” is concerned: categorizing living things and natural foods correlates with the left arcuate segment, left inferior fronto-occipital fasciculus and anterior corpus callosum bilaterally (pFWE < 0.05, see Fig. 2 for coordinates and details). See Fig. 2, for results of the VBM and TBSS conjunction analyses in the word-picture matching and categorization tasks.

For all tasks, results from DTI tractography analyses are consistent with those from the TBSS (see Supplementary Information S12 for details, and Fig. 2 as examples of the tracts involved).

The ***Sensory-functional matching task*** was not subjected to a conjunction analysis because it does not contain all four variables of interest but only natural and transformed food, and has been designed to specifically test the association between these two food types with sensory and functional properties. While we failed to find any supra threshold correlation for the sensory block, we found that accuracy at trials with transformed foods correlated with a large cluster in the right temporal cortex (bilateral PHG, right FG, temporo-occipital division of the MTG) and right OFC, regions implicated in processing the energetic value, that is a functional feature of food. The white matter tracts identified are consistent with the findings from this task and from other tasks as well. Specifically, we found an involvement of the right corpus callosum, anteriorly, for “sensory” trials, whereas for “functional” trials, accuracy correlated with a cluster in the left inferior longitudinal fasciculus (*p*FWE < 0.05) (see Fig. S6 in the Supplementary Information for details and MNI coordinates).

For all tasks, results from DTI tractography analyses are consistent with those from the TBSS (see Supplementary Information S12 for details).

#### Association between food recognition and i) gray matter volume and ii) fractional anisotropy

**Figure 3:**
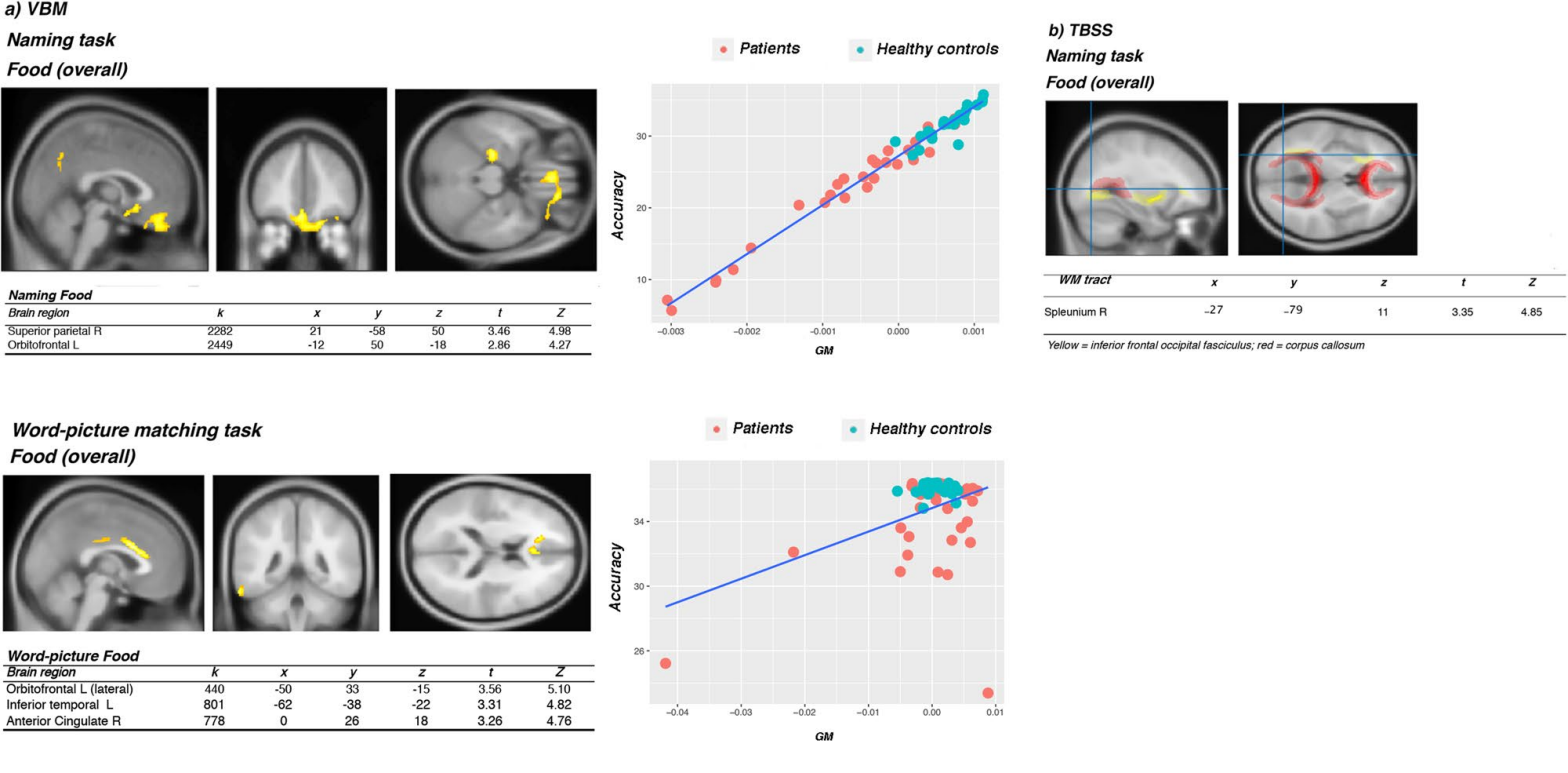
VBM (**a**) and TBSS (**b**) results for food overall, at the naming and the word-picture matching tasks. For the TBSS (**b**), the crosshair represents the coordinates corresponding to the peak cluster returned by the analysis. Images have been reconstructed as reported in the Methods section. All results reported have survived FWE correction (p FWE < 0.05). K = cluster size (number of voxels). The insets show scatter plots obtained by correlating the GM volume for each of our participants’ scans at that cluster with the behavioural variable of interest. On the y axis accuracy is reported, on the x axis GM values or tracts’ volumes are reported.

We ran multiple regression models, for each of the tasks, to correlate voxel-wise GM density and all participants’ accuracy on food, overall. Whereas accuracy scores for food at the ***Categorization*** did not correlate significantly with local GM density, the analysis on the food ***Naming*** and ***Word picture matching*** accuracy returned significant correlations with the OFC, for both tasks. Specifically, ***naming food*** accuracy correlated with a bilateral cluster in the OFC and inferior temporal cortex (ITC), and in the medial posterior cingulate cortex (PCC) (see Fig. 3), with the TBSS analysis showing a correlation with FA in the area subjacent the calcarine fissure and in the splenium of corpus callosum, bordering the inferior frontal occipital fasciculus (*p* FWE. < 05). The involvement of the left cingulum (*r* = 0.403, *p* = 0.004) also emerged in the DTI tractography analyses. For what concerns the ***Word picture matching***, beyond the involvement of left OFC, a second significant cluster emerged in the anterior cingulate cortex (see Fig. 3).

#### Semantic knowledge

**Figure 4:**
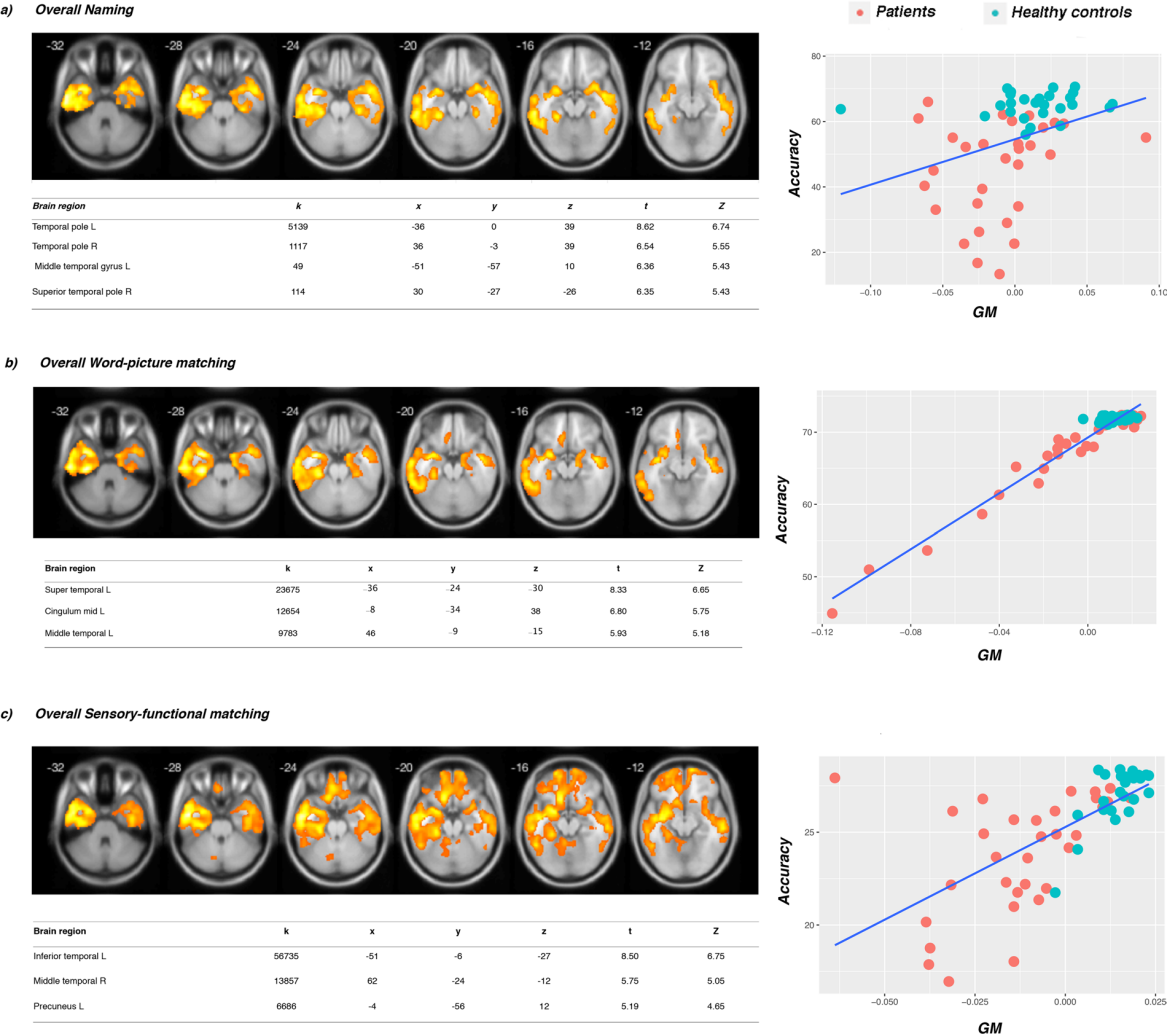
Semantic performance overall. (**a**) Shows significant clusters for the naming task, (**b**) clusters for the word-picture matching task and (**c**) the sensory-functional task (pFWE < 0.05). Correlations between categorization performance did not survive FWE correction, but we found a large cluster (k > 1000, p uncorrected < 0.001) in the right temporal cortex (x = 42, y = −10, z = −30). K = cluster size (number of voxels). The insets show scatter plots obtained by correlating the GM volume for each of our participants’ scans at that cluster with the behavioural variable of interest. On the y axis accuracy is reported, on the x axis GM values or tracts’ volumes are reported.

Finally, we correlated, with VBM, overall semantic performance with the local grey and white matter concentration, with the purpose of investigating the correlates of non category-specific semantic knowledge. For the ***naming*** task, we observed large bilateral clusters in the ATL, especially in the ventral portion. Similar results were observed in the ***word-picture matching task***, with a large cluster including the left temporal lobe extending to the FG. For what concerns **categorization**, we found a large cluster (k > 1000, *p* uncorrected < 0.001) in the right temporal cortex (x = 42, y = −10, z = −30), but it did not survive the FWE correction. The overall accuracy scores on the ***sensory-functional matching task*** correlated with a large cluster in the temporal lobe and a cluster peaking in the left precuneus an extending to the OFC, given that in this task only food stimuli were used (see Fig. 4 for details and coordinates).

## Discussion

In this study, we assessed whether the knowledge about natural food and living things, and knowledge about transformed food and nonliving things, is organized according to sensory and functional properties, as posited by the sensory/functional hypothesis (SFH^1,2,17,18^). Our results across different tasks are in accordance with the SFH, with correlations being found between atrophy of ‘sensory’ regions and reduced ability to recognize natural food and living things, on the one hand, and between atrophy of ‘functional’ regions and reduced ability to recognize transformed food and nonliving things, on the other. In the following, we will explain this pattern of VBM, TBSS and DTI tractography results.

### Natural food and living things: the “Sensory Knowledge”

One of the main results that emerged from the VBM analysis is that the reduced ability to recognize natural food mainly correlates with atrophy in sensory regions of the cortex such as LOC, cerebellum and the ITC, which have been repeatedly found associated to sensory processing (see^19^). The anterior corpus callosum (CC) turned out to be also involved in natural food recognition based on TBSS results. This tract is implicated in semantic processing and in sensory-motor tasks (for a review see^20^). We argue that the involvement of this tract might depend on natural food still needing to be prepared, as also suggested by a recent EEG study (Coricelli *et al*., under review^12^). In this latter study with healthy individuals, a peak in the waveform was observed as early as 130 ms from visual onset of natural food stimuli in the premotor cortex. This region not only is a primary sensory area, but is also the site of the somatic motor and sensory homunculus (Penfield & Boldrey^21^). Recently Catani^22^ revised Penfield’s view and showed that in this brain region there are co-localization of sensory maps representing different body parts, rather than a full segregation and critically between the hand region and mouth region. This might be due to the fact that hand-to-mouth movements are necessary to bring food to the mouth, and this is more likely to be associated with natural foods. Indeed, natural food is generally perceived as less readily edible than transformed food^23^.

Results from the VBM conjunction analysis, exploring recognition of both natural food and living things in the experimental tasks, confirm that the lexical-semantic information common to these stimuli is represented in sensory regions (LO, cerebellum, bilateral FG and ITC). The TBSS conjunction analysis investigating lexical-semantic processes of both natural food and living things, showed an involvement, among other tracts, of the inferior frontal occipital fasciculus, that connects the anterior temporal lobe with the OFC. This result, supported by DTI tractography analyses, highlights the involvement of this higher order region, involved in assigning a behavioural value to a stimulus (e.g., a plant).

### Transformed food and nonliving things: the “Functional Knowledge”

On the other hand, recognition of transformed food is mainly affected by atrophy in functional associated regions (e.g., left pMTG, AG, and STG among the others, see^13,14,19^), as well as regions associated with processing images of palatable or highly caloric foods (e.g. insular cortex, see^24^). The TBSS highlighted the involvement of the right cingulum (a bilateral correlation was observed but did not survive FWE correction), a tract that has been associated with high calorie food images processing (e.g.^25^), and the corpus callosum, which is involved in motor function (see^20^), in ideomotor apraxia in patients with Alzheimer’s disease^26^, as well as in transfer of semantic information^20^. Information about nonliving entities and transformed foods together (conjunction analysis) is coded in functional related regions (IFG, STG, MTG, posterior parietal cortex). Moreover, the sensory-functional matching task showed that performance in trials involving functional words and transformed food correlated with pMTG and STG, thus supporting to the hypothesis that transformed food shares features with utensils^9,13–15^. Moreover, performance on these trials also correlated with atrophy of the PHG (bilaterally) and right ITC, regions previously associated with high calorie food processing^24^. This latter result additionally suggests that the energetic content might be a relevant functional property in transformed food recognition. The TBSS conjunction analysis revealed that the left arcuate posterior segment and the right cingulum correlate with the recognition of transformed food and nonliving things together, across different tasks. In addition, we found a consistent involvement of the IL fasciculus with TBSS analyses, This white matter tract encompasses the regions we found involved in functional knowledge with the VBM analysis (MTG, STG).

Recognition of nonliving things correlates with atrophy of the precuneus cortex, previously associated with knowing about tools^13^, and of the STG^14^. Consistently, the TBSS highlighted the involvement of the corticospinal tract bilaterally, the corpus callosum (e.g.^27,28^), and the left inferior longitudinal fasciculus, involved in language processing (e.g.^29^) and encompassing the aforementioned MTG and STG.

The present findings strongly support the initial hypotheses that food breaks down in natural and transformed foods, and that this distinction depends upon a sensory/functional organization of semantic memory. Interestingly, behavioural results of the sensory-functional task, that was intentionally designed to test the sensory-functional hypothesis using food as a test category, show that bvFTD and PPA patients, but not Alzheimer’s disease patients, performed better in trials where sensory words are paired with natural food, probably due to the greater impairment at correctly categorizing natural food in the latter group (see S6 for details). Moreover, these results are in line with recent studies, supporting the relevance of the natural/transformed food distinction^30–32^. In particular support of the hypothesis that the natural/transformed distinction relies on sensory/functional properties, a recent ERP study, found that, in normal weight individuals, the N400 component, usually modulated by semantic or syntactic incongruency, was larger for sensory primes paired with transformed foods and functional primes paired with natural foods^12^. Moreover, it seems that the brain is able to track the difference between natural and transformed food as early as around 130 ms (Coricelli *et al*., under review).

### A possible ‘food network’

Our findings allow us also to identify several brain regions that appear to be strongly involved in semantic knowledge about food. Specifically, patients’ performance with food correlates with OFC, ITC - especially the posterior fusiform cortex – and LOC and the white matter tracts connecting these regions, such as the IFOF, connecting occipital areas with the OFC. One could speculate that all these regions (OFC, ITC, LO) may together constitute a network responsible, together with the temporal lobes, of processing information concerning food and, by assigning a value to the stimulus (OFC, e.g.^33^), to plan how to act upon it (see for instance^34,35^). In fact, the IFOF puts temporal and frontal regions in communication. This result suggests that food may overall be processed as an extremely salient stimulus thanks to both sensory and functional properties.

Another interesting result concerns transformed food. Indeed, behaviorally, transformed food was overall named significantly better than natural food, consistently with other studies showing an advantage for this category^30–32^. Another interesting result is that only bvFTD patients named food significantly better than non-food and that their performance, particularly with transformed food, significantly correlates with calorie content (see Supplementary information S3b). This has been not observed with any of the other groups. This result is interesting and deserves to be further investigated since bvFTD is known to cause several eating abnormalities, among which a strong preference for foods that are palatable and high in calorie content (for a review see^24^).

### Towards a comprehensive model of semantic memory

Taken together, our results highlighted a set of cortical regions and white matter tracts involved in semantic processing, and add evidence on current models of semantic memory. They confirm and extend the SFH theory, since they show a set of regions (e.g. bilateral OFC, FG), connected by white matter tracts (e.g. the IFOF), that are associated with sensory knowledge, and a set of cortical regions (IFG, STG, MTG, posterior parietal cortex), connected by white matter tracts (e.g. the inferior longitudinal fasciculus), associated with functional knowledge.

The existence of these sets of regions suggests that objects may be primarily recognized based on properties (e.g., visual, olfactory, gustatory vs. functional, motor) that are more diagnostic for their recognition and that these properties are likely to be organized in two main subsystems (sensory and functional). We speculate that the sensory vs. functional initial information is eventually integrated in the ventral ATL, bilaterally, that has been proposed to be pan-modal hub (see for instance^5^). Consistently with this view, in our study, the vATLs emerged only when considering the semantic performance overall. This result is supported both behaviourally and anatomically. PPA patients do not show any category-specificity in their semantic deficit: this suggests that the ATLs, strongly and almost selectively damaged in these patients, are organized in an a-modal structure. This is supported also by the massive involvement, across categories, of the inferior longitudinal fasciculus, especially on the left side. This white matter tract has been suggested to connect ventral aspects of the ATL with other temporal and dorsoparietal regions^36^. In our view, our results from both TBSS and VBM suggest that ATLs may be critical in integrating semantic information. Our results are also in line with a recent model emphasizing the role of the ATLs as a semantic panmodal hub, as the C^3^ model^5^ according to which visual features are critical for recognizing living things, and vATLs represent cross-modal hubs communicating with modality-specific spokes (see^6,7^). Our results converge with the view that ATLs integrate semantic information.

In conclusion, our results strongly support a sensory-functional organization of semantic knowledge, with information concerning food breaking down in natural and transformed and being stored in sensory versus functional brain regions, respectively, rather than in a domain-specific fashion.

## Materials and Methods

### Participants

Fifty-eight right-handed participants took part in the study: ten bvFTD patients (five females), eleven PPA patients (four females), eleven Alzheimer’s disease patients (six females), and twenty-six healthy controls (sixteen females). Specifically, the diagnosis of Alzheimer’s disease followed the DSM-V (American Psychiatric Association, 2013) criteria, of bvFTD that of Neary *et al*.^37^ and of PPA the criteria outlined by Noppeney *et al*.^38^, that include also anomia and semantic impairments. Participants were admitted to the study if they had at least five years of formal education, were native speakers of Italian, had no visual or hearing deficits, had no history or evidence of cerebrovascular diseases, major psychiatric disorders, brain tumour, and alcohol or drug abuse. Participants were matched for gender (χ^2^ = 0.82, *p* = 0.85), age and education (*ps* = ns) (see Supplementary Information S1a). Patients were diagnosed by the neurologists that took part in the study (R.A., C.T., M.G), based both on neurological and neuropsychological evaluations. The MMSE^39^, Addenbroke’s Cognitive Examination revised^40^, the Frontal Assessment Battery^41^ were administered to all participants; in addition, patients were administered a naming task^42^. Moreover, when possible, also the Pyramid and Palm Trees test (see^43^) and the Camden recognition memory test for faces^44^ were administered (see Supplementary Table S1b). Participants read and signed an informed consent. The study was conducted in accordance to relevant guidelines (Helsinki declaration, 2013) and approved by the SISSA ethics committee.

### Experimental tasks

Participants were administered with four behavioural experimental tasks following the same order: categorization task, confrontation naming, word-picture matching and the sensory/functional matching task. Their responses were recorded by the experimenter on a scoring sheet. The order of items was first randomized within each task and then administered with the same order across participants. Tasks were presented on a computer screen by using Microsoft Powerpoint presentations. Before each task, participants performed an 8-trial practice in order to familiarize with the task, and these responses were not included in the analyses.

#### Task 1–Naming

This task tests the ability to retrieve the names of visually presented pictures. Each of 72 pictures was presented individually at the centre of the computer screen and participants were asked to name it in a self-paced way. The 72 pictures where divided in: 18 natural foods, 18 transformed foods, 18 living things, 18 nonliving things. The confrontation naming detects the presence of eventual perceptual, lexical or semantic deficits, allowing us to detect a semantic deficit for one or more of the categories of interest.

#### Task 2–Word-picture matching

This task aims at evaluating the participants’ ability to understand nouns. In each of the 72 trials, the participant is required to point the target picture corresponding to the word uttered by the experimenter among five distractors. One of the five distractors is an exemplar of the same semantic category, whereas the other four distractors have been selected randomly. This task evaluates the lexical-semantic processing of words and, compared to the confrontation naming, it does not require a verbal output.

#### Task 3–Categorization

This task evaluates the participants’ ability to categorize stimuli. In each of 144 trials (organized in 4 blocks), two pictures were presented, next to each other. Participants were asked to decide whether the two items belonged to the same category or to different categories. In the same trials, two foods or two non-foods were depicted, whereas in the different trials, one food and one non-food item. The four blocks were divided in: natural food and living things; natural food and nonliving things; transformed food and living things; transformed food and nonliving things. This configuration of trials allows assessing whether there is an impairment in a specific category (same trials), but also whether for participants it is more difficult to judge natural foods as different from living things rather than from nonliving things, or transformed foods as different from nonliving things rather than from living things.

#### Task 4–Sensory/functional matching task

This task was specifically designed to test whether pairing a word representing a sensory property with a natural food, and pairing a word describing a functional property with transformed food (congruent trials) would improve accuracy compared to trials in which natural food and transformed food were paired with functional and sensory properties, respectively (incongruent trials). Specifically, participants were asked to decide whether the word paired with the image was true or false with respect to that image (e.g. lasagne – lunch; almonds – hard). In addition, for each item we presented two filler trials, in which incorrect congruent and incongruent pairings (e.g. cauliflower – sweet, lasagne – breakfast) were presented, in order to avoid participants to respond always in the same manner to the question. Such trials were not considered for the behavioural analyses. The task (56 trials) consisted in two blocks, one pairing words describing sensory features (e.g. ‘sweet’) and the second block pairing words describing functional features (e.g. ‘lunch’) with either natural or transformed foods. The best congruent and incongruent pairings, as well as the most suitable words, were selected with a pilot study (see Supplementary Information S2).

### Stimuli

For Tasks 1–3 seventy-two images depicting food and non-food items were selected from FRIDa database^22^, with a resolution of 530 × 530 pixels and colour coding RGB. Non-food stimuli were both living (N = 18) and nonliving things (N = 18), and food stimuli both natural (N = 18) and transformed foods (N = 18). Stimuli were matched for letter length, written frequency and discriminability across all four categories^45^. For Task 4, 14 images of food stimuli only were selected (FRIDa^23^), with seven natural foods and seven transformed foods, matched for number of letters, written frequency, arousal, valence, familiarity, typicality and discriminability. Natural and transformed food stimuli were matched for calorie content (for details on psycholinguistic variables and property ratings see SI 1).

### Data analysis

#### Behavioural analyses

Patients divided in three groups (bvFTD, PPA and Alzheimer’s disease) were compared in both demographical and clinical measured to healthy controls, through independent samples t-test or, when necessary, Mann Whitney U tests (See Supplementary S2a,b).

Performance on the naming task was analysed using a repeated-measures ANOVA with *Group* (AD, bvFTD, PPA, healthy controls) as between-subjects factor, *Type of item* (food, non-food) and *level of artificiality* (sensory, functional) as within-subject factors. We explored significant interactions with the analysis of simple effects (see^46^). Results are described in the Supplementary Information S3. Performance on the categorization task and the word-picture matching task was analysed using the Mann-Whitney tests, as data were not normally distributed. Differences between food and non-food and natural and transformed food within each of the groups were analyzed using the Wilcoxon rank sum tests. Results from these latter three tasks are reported in the Supplementary Information S4 and S5. Performance on the sensory-functional matching task was analysed with a repeated-measures ANOVA with *Group* as between-subjects factor, *Type of Food* (natural, transformed) and *type of property* (sensory, functional) as within-subject factors (see S6). Multiple comparisons were corrected with Bonferroni for all the analyses. Finally, regression analyses were conducted between naming performance and caloric content, separating the patients in the three groups, concerning food items and significant results are reported in S3.

Analyses were performed with statistical software PASW Statistics for Windows, Version 18.0. Chicago, SPSS Inc. Scatter plots were produced using the ggplot2 package in R^47^.

#### Anatomical analyses

Voxel Based Morphometry: Scanning sessions were carried out in order to acquire T1-weighted MRI images for each participant. Images were acquired with a Philips Achiva 3 T scanner in Ospedale S. Maria della Misericordia in Udine. Images were collected using T1W3D TFE SENSE rapid gradient fast field echo sequence (TR/TE = 8.1/3.7 msec), with a 8.1 degree flip angle, saggittal orientation perpendicular to the double spin-echo sequence, 1 × 1 × 1 mm in-plane resolution, with 170 mm slab thickness.

Voxel Based Morphometry (VBM^48^) was used to correlate local grey matter concentration with our variables of interest, which are participants’ accuracies in the experimental tasks. Analyses were performed with SPM12 software package. First, the T1-weighed structural images were reoriented by setting the origin at the anterior commissure for each of the subjects. Subsequently, the preprocessing was carried out by segmenting the images in grey matter, white matter and cerebrospinal fluid. Grey and white matter segments of our participants were then used, with DARTEL tools, to create a template brain, which consisted in an average of the brains (segmented grey and white matter) of our participants. After this step, the grey matter images obtained for each of the subjects after the segmentation step were co-registered to the template and normalized to the MNI space and smoothed with a 8-mm isotropic Gaussian kernel.

Once completed the preprocessing, a covariates only statistical model was applied, with gender and age as nuisance covariates, group as a factor, and accuracy on the experimental tasks as covariates of interest. This model allows to enter all subjects as a single group, independently of their diagnosis, and to take into account individual differences in the pattern of atrophy and of cognitive decline (for a discussion see^49^; see also^50^). Moreover, to identify the brain regions that correlated with the scores on two categories together conjunction analyses were also performed^51^. Thus, in the ‘results’ section, we selected the two contrasts of the conjunction analysis with which we aimed to search for possible overlaps (see, for instance^52,53^). In particular, we selected “living things + natural food” performance, and “non-living things + transformed food” performance. We applied this procedure to each of the semantic tasks. Total intracranial volume for each participant was used as global normalisation in the analysis, for which we chose the ANCOVA option. Images were produced using Xjview^54^.

Additionally, we extracted the Y variable for the significant clusters resulting from each of the analyses on the semantic categories of interest, in order to obtain an approximation to the mean volume inside that cluster for each of the participants. The scope was to use that value to run further correlational and regression analyses. Some of the results are reported as scatter plots in Figs 1–4 of the main text. Further results are reported in S9, and provide additional correlations between functional/transformed semantic performance and GM volumes across tasks.

We computed VBM multiple regression models for each of the patients’ group separately, in order to investigate whether there was a correspondence between the accuracies at each test and their brain atrophy. These analyses are reported in the Supplementary Information S10. Finally, we ran separate VBM within-subject ANOVAs for each of the patient groups, compared to healthy controls, in order to explore whether the pattern of atrophy observed matched the diagnosis and regions associated with the semantic tasks. Results are reported in S11.

#### Tract based spatial statistics

Diffusion tensor data were acquired using an axial diffusion-weighted single-shot spin-EPI sequence covering the whole brain (TE/TR = 9110/71 m, bandwidth = 1287 Hz/pixel, flip angle = 90, 57 contiguous axial slices, 2 mm slice thickness, head 8channel coil, SENSE factor 2). Two b values were used (b = 0, b = 800); 1 image at 0 sec/mm^2^ (no diffusion weighting) and 64 non-coplanar images at 1000 sec/mm^2^ (diffusion-weighting b value) were acquired. The gradient directions were uniformly distributed on a sphere.

The analysis of the diffusion tensor imaging data was performed using the FMRIB diffusion toolbox^55^. First we generated binary masks for each of the images of our participants, by means of the brain extraction tool (version 4.1), with fractional threshold = 0.1 and vertical gradient, g = 0. Subsequently, we performed the eddy current correction on the original images of each participant; then, using the brain extracted mask and the b value and the b vector, fractional anisotropy (FA) for each participant was obtained.

Upon completion of the preprocessing, the brain extracted FA images of all participants were used as the input images for TBSS processing^55^. First, we performed a voxelwise nonlinear registration of all participants’ FA to a standard image provided by the FSL software. The FA images obtained in this way were, then, averaged to create a mean FA image, which was subsequently used to create a skeleton of the tracts of white matter. This procedure labels the skeleton voxels with maximum FA intensity along the perpendicular direction of the tract; we used a FA threshold of 0.3 to distinguish grey from white matter^56^. Subsequently, the mean skeleton was applied to the registered FA image of individual participants. Then, we projected the maximum FA values on the skeleton for our subsequent statistical analysis by using the SPM basic models function to design a covariates only statistical model, mirroring the model used to analyse the VBM data (e.g.^57^). In order to identify the white matter tracts involved, based on the statistical parametric map obtained, the diffusion tensor imaging-based atlases by Thiebaut de Schotten *et al*.^58,59^ and Oishi *et al*.^60^ were used.

In addition, the FA images obtained as previously described, were used also for DTI tractography with the purpose of exploring the correlations of the white matter tracts resulting from the TBSS analysis and the behavioural variables. Streamlines were reconstructed as described in^61^ and using the StarTrack toolbox^62^. Streamlines were propagated setting fractional anisotropy threshold to 0.2, with a step- size of 1 mm, and with Euler integration.

On FA images in the participants’ native space, we defined regions of interest based on the results of the whole-brain TBSS. We performed virtual dissections with TrackVis (www.trackvis.org ^63^) and extracted the volume (ml) of each tract of interest in all participants. These volumes of the tracts and the scores at the categories of interest for each significant TBSS result, were used to produce scatter plots (natural food, transformed food, living, nonliving, sensory/functional matching task). In addition, examples of reconstructed white matter tracts are reported, when relevant. Results are reported in the results’ figures and, when appropriate, in the Supplementary information (S7, S8, S12).

For both TBSS and VBM analyses, we reported clusters and peaks at significance level *p*FWE < 0.05.

## Supplementary information

Supplementary Information revised
