# Supplementary material for: Food knowledge depends upon the integrity of both sensory and functional properties: a VBM, TBSS and DTI tractography study
Source: Sci Rep. 2019 May 15;9:7439. doi: 10.1038/s41598-019-43919-8 (PMC6520382; doi:10.1038/s41598-019-43919-8)
Supplement: Supplementary file 1 — Supplementary Information revised [file 41598_2019_43919_MOESM1_ESM.docx]

**Supplementary information**

**Food knowledge depends upon the integrity of both sensory and functional properties: a VBM, TBSS and DTI tractography study**

Miriam Vignando^1^, Marilena Aiello^1^, Adriana Rinaldi^2^, Tatiana Cattarruzza^3^, Giulia Mazzon^3^, Paolo Manganotti^3^, Roberto Eleopra^2^ & Raffaella I. Rumiati^1,4^.

1. Neuroscience and Society Laboratory, SISSA, Trieste, Italy

2. Department of Neurology, "S. Maria della Misericordia" University Hospital, Udine, Italy.

3. Department of Medical, Surgical and Health Sciences, Clinical Neurology Unit, Cattinara University Hospital, Trieste, Italy

4. ANVUR, Italy

**Supplementary Information**

**S1**. *Description of the Pilot study for the Sensory/functional matching task.*

The first block (N trials = 28) included either natural or transformed foods, each of which was paired with a word describing a sensory property (‘sweet’, ‘salty’, ‘soft’, hard’). Sensory properties depicted taste or texture (see also 27, 28). In the second block (N = 28), either natural or transformed foods were presented, and each of them was paired with a word describing a functional property We defined as functional properties of food the context (‘lunch’, ‘break’), the status (‘cheap’, ‘expensive’) and the healthiness (‘unhealthy’). As for the sensory block, for each of the items, a congruent and an incongruent trial was presented in half of the trials. In order to use only words properties that were only sensory in the sensory block and related only to function in the functional block, we carried out a pilot study on 20 young healthy participants. Participants were asked to rate, on a Likert scale ranging from 1 to 7, whether a word depicted a sensory or a functional property and we chose as sensory only those words that were rated as highly sensory and weakly functional (p < .001) and as functional those words that were rated as highly functional and weakly sensory (p < .001) (see S1a). In addition, stimuli were matched for written frequency, number of letters, calorie content, arousal, valence and familiarity (see S1b).

**a)** Pairwise comparisons on sensory and functional ratings attributed to each of the adjectives. Only adjectives rated high in “sensoriality” and low in “functionality”, and viceversa, were selected.


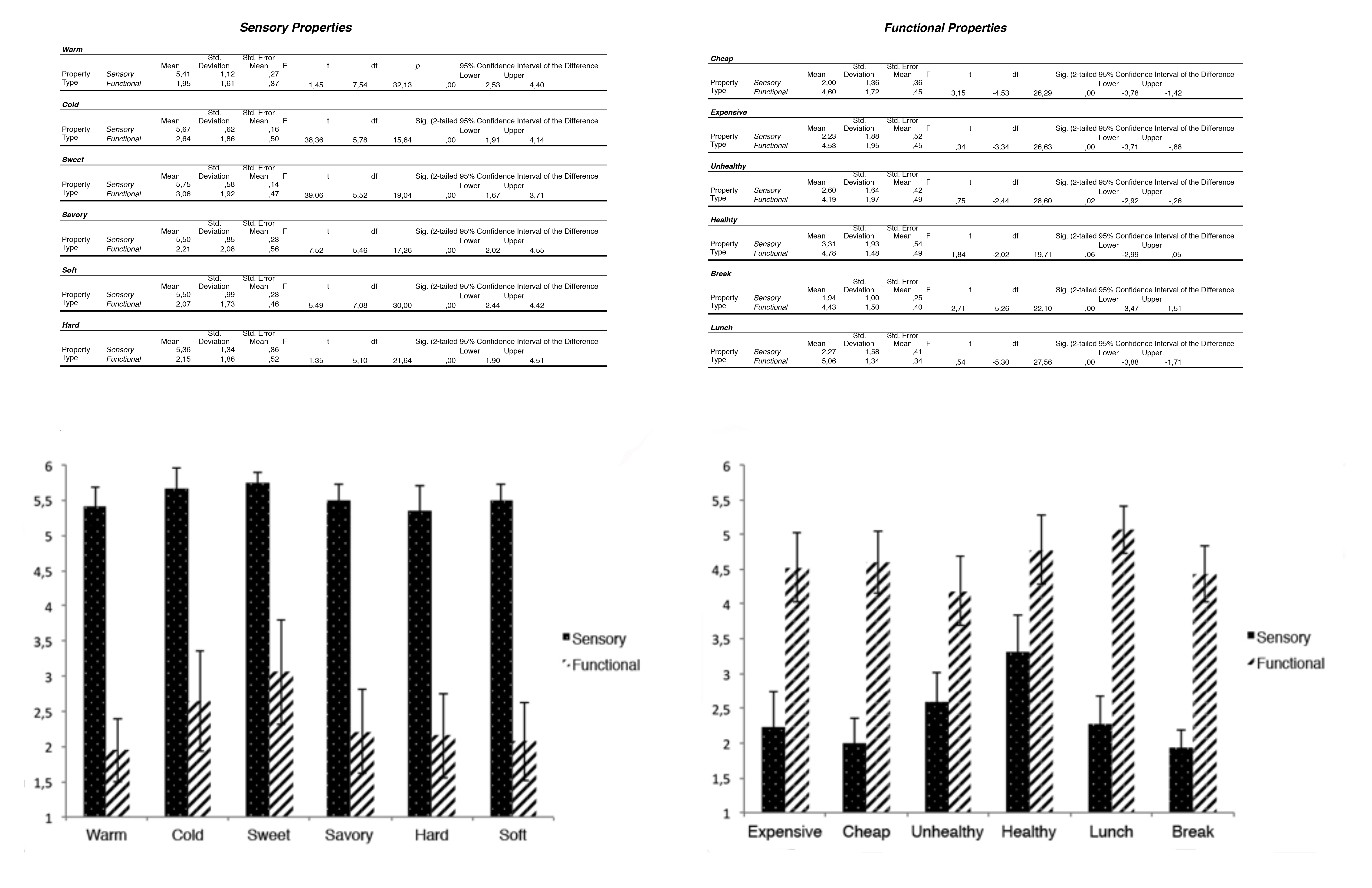


*b) Relevant psycholinguistic and visual variables for which Task 4 is matched. The only variable at which natural vs. transformed foods differ significantly (p <. 01) is the level of transformation, which was intentional for the purpose of the study. All other variables are matched (p > .05).*

| **Natural Foods** |  |  |  |  |  |  |  |  |
| --- | --- | --- | --- | --- | --- | --- | --- | --- |
| *Item name* | **Calories** | **Immediate edibility** | **Transformation** | **Valence** | **Familiarity** | **Typicality** | **Discriminability** | **Arousal** |
| chestnut | 31.76 | 40.96 | 9.24 | 59.11 | 45.41 | 69.15 | 19.45 | 26.84 |
| coconut | 35.38 | 26.88 | 7 | 76.24 | 34.05 | 80.14 | 5.35 | 49.45 |
| dates | 50.19 | 6.33 | 16.75 | 46.3 | 33 | 73.1 | 18.16 | 28 |
| almonds | 69.05 | 12.71 | 11.96 | 65.55 | 57.44 | 82.48 | 9.1 | 29.92 |
| mussel | 37.54 | 42.84 | 16.65 | 56.65 | 33.89 | 64.42 | 8.4 | 36.5 |
| cauliflower | 9.6 | 57.5 | 6.54 | 58.3 | 53.61 | 80.59 | 8.8 | 27.81 |
| shrimp | 43.93 | 67.8 | 8.15 | 63.86 | 40.96 | 57.35 | 8.5 | 36.88 |
| oyster | 33.85 | 42.17 | 14.28 | 50.24 | 23.05 | 46.58 | 28.77 | 40.42 |
|  |  |  |  |  |  |  |  |  |
|  |  |  |  |  |  |  |  |  |
| **Transformed Foods** |  |  |  |  |  |  |  |  |
| *Item name* | **Calories** | **Immediate edibility** | **Transformation** | **Valence** | **Familiarity** | **Typicality** | **Discriminability** | **Arousal** |
| cracker | 63.86 | 5.7 | 49.48 | 57.3 | 51.95 | 71.14 | 5.9 | 33.54 |
| french fries | 75.35 | 14.77 | 41.96 | 53.5 | 50.37 | 69.8 | 9.05 | 41.35 |
| meatballs | 66.48 | 17.71 | 70.3 | 59.32 | 46.5 | 65.76 | 32.67 | 35.72 |
| nougat | 82.62 | 7.65 | 65.54 | 65.08 | 40.25 | 73.46 | 5.85 | 29.43 |
| octopus | 48.05 | 69.8 | 16.71 | 49.55 | 24.72 | 65.38 | 12.82 | 47.14 |
| risotto | 67.15 | 12.76 | 71.6 | 64.57 | 45.14 | 54.85 | 17.19 | 50.76 |
| pickles | 22.44 | 19.63 | 10.13 | 31.96 | 31.09 | 43.86 | 11.8 | 19.15 |
| courgettes | 33.62 | 11.56 | 45.5 | 74 | 61.08 | 84.96 | 5.12 | 41.96 |

**S2. Demographical information and neuropsychological assessment**

**a) Demographical information.** A one-way ANOVA was carried out, with *Age* and *Education* as dependent variables and *Group* as factor. Neither Age [*F*(3,55) = 1.72 , *p* = .173], nor Education [*F*(3,55) = 1.49 , *p* = .29] resulted as significantly different.

|  |  | **Age** |  |  |  | **Education** |  |
| --- | --- | --- | --- | --- | --- | --- | --- |
|  | Mean | Std. Dev | SEM |  | Mean | Std. Dev | SEM |
| **bvFTD** | 72.90 | 4.77 | 1.51 |  | 9.70 | 4.06 | 1.28 |
| **AD** | 73.91 | 6.41 | 1.93 |  | 9.73 | 4.10 | 1.24 |
| **PPA** | 73.18 | 6.31 | 1.90 |  | 10.36 | 5.39 | 1.63 |
| **HC** | 70.67 | 5.37 | 1.03 |  | 11.89 | 3.88 | 0.75 |

**b)** **Neuropsychological assessment**. Patients were all outperformed by healthy controls at the MMSE [specifically, bvFTD (*t* _10.54_= -3.15, *p* < .001), Alzheimer’s disease (*t* _12.39_= -5.75, *p* < .001), PPA (*t* _10.68_= -4.61, *p* = .001)] and the FAB [specifically, bvFTD (*t* _10_= -4.9, *p* = .001), Alzheimer’s disease (*t* _11.78_= -4.77, *p* < .001), PPA (*t* _10.10_= -5.90, *p* < .001)].Moreover, bvFTD show a higher MMSE score with respect to PPA patients (*t* _16.7_= 2.2, *p* =.04).

bvFTD patients performed worse than controls at each of the ACE-R subscales administered (*p*s <.05), except for the subscale of the ACE-R testing language (*t* _9_= -1,7, *p* = .12). Alzheimer’s disease patients did not perform significantly worse than controls only at the ACE-R visuospatial subscale (*t*_10_ = -1.1, *p* = .3), whereas PPA patients performed worse than controls at all of the subscales (*ps* < .001). bvFTD patients performed better than Alzheimer’s disease patients only at the ACE-R memory subscale (*t*_19_ = 2.2, *p* = .04), better than PPA patients at the fluency (*t* _19_= 2.6, *p* = .02), language (*t*_19_ = 3.7, *p* = .002), visuospatial (*t* _19_= 2.8, *p* = .01) and total (*t* _19_= 2.9, *p* = .01) scores of the ACE-R. Moreover, PPA performed worse than AD at the subscales of fluency (*t* _20_= 2.6, *p* = .02) and language (*t* _12_= 4.7, *p* = .001), and also at the total score (*t* _20_= 2.11, *p* = .05).

We also compared patients only at the Pyramid and Palm Trees Test, at the Camden test and at a picture naming. However, not all of our patients were able to complete these tests (see table 1). bvFTD and Alzheimer’s disease patients did not differ significantly at any of these tests (*p*s > .05), whereas PPA patients performed significantly worse than bvFTD (*t* _10_= 4.25, *p* = .002) at the PPTT. Moreover, PPA patients’ naming performance (Laiacona) was significantly worse than both bvFTD (*U* = 10.5, *p* = .03) and Alzheimer’s disease (*U* = 7.0, *p* = .02). For details, see the table below (in the table, Alzheimer’s disease is shortened as AD and healthy controls as HC).

| ***Neuropsychological assessment: patients and healthy controls*** | | | | |  |
| --- | --- | --- | --- | --- | --- |
|  |  |  |  |  |  |
|  | **Group** | **N** | **Mean** | **Std. Dev** | **Std. Error Mean** |
| **MMSE** | bvFTD | 10 | 25.79 | 4.00 | 1.27 |
|  | AD | 11 | 22.95 | 3.38 | 1.02 |
|  | PPA | 11 | 20.61 | 6.60 | 1.99 |
|  | HC | 26 | 29.96 | 1.73 | 0.34 |
| **ACE_orientation** | bvFTD | 10 | 15.80 | 2.57 | 0.81 |
|  | AD | 11 | 14.18 | 2.04 | 0.62 |
|  | PPA | 11 | 11.91 | 5.52 | 1.66 |
|  | HC | 26 | 18.00 | 0.00 | 0.00 |
| **ACE_memory** | bvFTD | 10 | 14.00 | 5.98 | 1.89 |
|  | AD | 11 | 8.91 | 4.37 | 1.32 |
|  | PPA | 11 | 10.36 | 7.21 | 2.18 |
|  | HC | 26 | 23.31 | 2.65 | 0.52 |
| **ACE_fluency** | bvFTD | 10 | 6.40 | 2.88 | 0.91 |
|  | AD | 11 | 6.18 | 2.52 | 0.76 |
|  | PPA | 11 | 3.18 | 2.79 | 0.84 |
|  | HC | 26 | 11.65 | 2.13 | 0.42 |
| **ACE_language** | bvFTD | 10 | 23.40 | 4.22 | 1.33 |
|  | AD | 11 | 24.09 | 1.97 | 0.59 |
|  | PPA | 11 | 14.82 | 6.26 | 1.89 |
|  | HC | 26 | 25.73 | 0.45 | 0.09 |
| **ACE_visuospatial** | bvFTD | 10 | 14.10 | 1.45 | 0.46 |
|  | AD | 11 | 14.18 | 5.34 | 1.61 |
|  | PPA | 11 | 10.27 | 4.20 | 1.27 |
|  | HC | 26 | 15.92 | 0.39 | 0.08 |
| **ACE total** | bvFTD | 10 | 72.70 | 13.94 | 4.41 |
|  | AD | 11 | 65.73 | 11.04 | 3.33 |
|  | PPA | 11 | 50.55 | 21.08 | 6.36 |
|  | HC | 26 | 94.81 | 4.56 | 0.89 |
| **FAB** | bvFTD | 9 | 12.00 | 2.74 | 0.91 |
|  | AD | 11 | 11.64 | 3.38 | 1.02 |
|  | PPA | 10 | 9.20 | 3.91 | 1.24 |
|  | HC | 26 | 16.73 | 1.10 | 0.28 |
|  |  |  |  |  |  |
| ***Neuropsychological assessment: patients only*** | | |  |  |  |
|  |  |  |  |  |  |
|  |  | **N** | **Mean** | **Std. Dev** | **Std. Error Mean** |
| **PPTT** | bvFTD | 7 | 48.14 | 4.06 | 1.53 |
|  | AD | 2 | 43.50 | 7.78 | 5.50 |
|  | PPA | 5 | 34.20 | 7.33 | 3.28 |
| **RMT** | bvFTD | 6 | 18.33 | 4.63 | 1.89 |
|  | AD | 8 | 16.25 | 2.71 | 0.96 |
|  | PPA | 5 | 17.40 | 2.70 | 1.21 |
| **NAMING (Laiacona)** | bvFTD | 10 | 58.50 | 5.38 | 1.70 |
|  | AD | 10 | 57.80 | 3.85 | 1.22 |
|  | PPA | 6 | 40.83 | 15.54 | 6.34 |
|  |  |  |  |  |  |

**S3.** ***Behavioural analysis on Naming task***

*a) ANOVA.* Thirty out of thirty-two patients performed this task (two patients were not able to complete it). The repeated measures ANOVA revealed *first*, a significant main effect of *Group* [*F*_(3, 53)_ = 41.93, *p* < .001, η2 .71], with healthy controls outperforming bvFTD (*p* < .001, 1.85, 6.05, 95% of CI), Alzheimer’s disease (*p* = .006, .54, 4.60, 95% of CI) and PPA (*p* < .001, 6.56, 10.94, 95% of CI) patients. PPA patients performed significantly worse than both Alzheimer’s disease (*p* <.001, -8.02, -2.04, 95% of CI) and bvFTD (*p* = .012, -7.37, -0.62, 95% of CI) patients, whereas these latter groups did not differ from each other. *Second*, we observed a significant main effect of the *Level of artificiality* [*F* _(3, 53)_ = 14.99, *p* < .001, η2 = .23], with artificial items (transformed food, nonliving things) being overall named better than natural entities (natural food, living things) (.37, 1.15, 95% of CI). *Third,* a significant *Type of item * Group* interaction was found [*F*_(3, 53)_ = 2.92, *p* = .04, η2 = .14], with bvFTD patients naming food significantly better than non-food, whereas the other groups did not show this difference. Finally, we found a significant interaction *Type of item * Level of Artificiality* [*F*_(3, 53)_ =23.42, *p* < .001, η2 = .31], with transformed food being named significantly better than natural food (*p* < .001, .56, 1.71 95% of CI), whereas the opposite pattern was observed for natural items, with nonliving things named worse than living things (*p* = .01, -1.53, -.21, 95% of CI).

*Below we report descriptive statistics for each of the groups.*

| ***Naming*** |  |  |  |  |  |
| --- | --- | --- | --- | --- | --- |
|  |  |  |  |  |  |
| ***bvFTD*** |  |  |  |  |  |
|  | **N** | **Min** | **Max** | **Mean** | **Std. Dev.** |
| Food | 10 | 21.00 | 32.00 | 25.60 | 3.78 |
| Nonfood | 10 | 13.00 | 31.00 | 23.60 | 6.90 |
| Natural food | 10 | 8.00 | 16.00 | 11.90 | 2.92 |
| Transformed food | 10 | 11.00 | 16.00 | 13.70 | 1.89 |
| Living | 10 | 5.00 | 16.00 | 11.40 | 3.63 |
| Nonliving | 10 | 6.00 | 16.00 | 12.20 | 3.49 |
|  |  |  |  |  |  |
|  |  |  |  |  |  |
| **AD** |  |  |  |  |  |
|  | **N** | **Min** | **Max** | **Mean** | **Std. Dev.** |
| Food | 11 | 22.00 | 32.00 | 27.82 | 3.43 |
| Nonfood | 11 | 18.00 | 34.00 | 26.91 | 4.50 |
| Transformed food | 11 | 11.00 | 17.00 | 14.55 | 2.34 |
| Natural food | 11 | 11.00 | 15.00 | 13.27 | 1.62 |
| Living | 11 | 9.00 | 17.00 | 14.00 | 2.37 |
| Nonliving | 11 | 9.00 | 17.00 | 12.91 | 2.30 |
|  |  |  |  |  |  |
|  |  |  |  |  |  |
| **PPA** |  |  |  |  |  |
|  | **N** | **Min** | **Max** | **Mean** | **Std. Dev.** |
| Food | 8 | 6.00 | 30.00 | 15.13 | 8.68 |
| Nonfood | 8 | 7.00 | 29.00 | 15.38 | 7.52 |
| Natural food | 8 | 1.00 | 14.00 | 6.50 | 4.84 |
| Transformed food | 8 | 4.00 | 16.00 | 8.63 | 4.03 |
| Living | 8 | 2.00 | 16.00 | 7.63 | 4.37 |
| Nonliving | 8 | 2.00 | 13.00 | 7.75 | 3.49 |

*b) We also ran regression analyses with calorie content as a predictor of naming performance. The model resulted significant for bvFTD patients onl [F(1,16) = 4.38, p = .05, Beta = .45], with the 46% of explained variance. None Alzheimer’s disease neither PPA patients’ naming performance was significantly predicted by caloric content.*

*c) Correlation analyses were carried out between naming performance and the psycholinguistic variables for which the stimuli of tasks 1-3 were not matched: familiarity, typicality, age of acquisition. As shown in the table below, these variables significantly correlate with naming performance of all groups, healthy controls included, and therefore the results of the VBM and TBSS analyses cannot be accounted by psycholinguistic variables.*

|  |  | *Naming accuracies per group* | | |  |
| --- | --- | --- | --- | --- | --- |
|  |  | **HC** | **bvFTD** | **AD** | **PPA** |
| **Familiarty** | r | .451** | .615** | .655** | .517** |
|  | p | <.001 | <.001 | <.001 | <.001 |
|  |  |  |  |  |  |
| **Typicality** | r | .333** | .417** | .434** | .394** |
|  | p | <.001 | <.001 | <.001 | <.001 |
|  |  |  |  |  |  |

**S4.** **Behavioural analysis on** **Word-Picture matching tas**k.

1. ***Mann Whitney U tests.***

*Comparisons between patients’ groups and healthy controls. bvFTD patients’ accuracy was lower than healthy controls’ at non-food (p = .002, U = 41), nonliving (p = .002, U = 41.5) and transformed food (p = .04, U = 65) recognition. Patients with Alzheimer’s disease performed worse than healthy controls only at trials assessing the ability to recognize non-food (p = .03, U = 69). PPA patients were impaired, with respect to controls, at all categories (all ps < .05).*

*Comparisons between patients’ groups. No difference was observed in bvFTD patients’ compared to Alzheimer’s patients (ps >. 05). When compared to PPA patients, the bvFTD group performed better than the PPA group in food recognition only (p = .05, U = 23.5). Alzheimer’s patients performed better than PPA at all categories (ps <. 05), except for living things (p = .2, U = 35.5).*

*It is to note, as the descriptive statistics table depicts, that all patients’ groups, even when performing significantly worse than healthy controls, did not show a great difference in the scores. Indeed, this task is less difficult than the naming task.*

1. ***Wilcoxon rank-sum tests and descriptive tables.***

*No significant category-specific effect was observed, except for a better performance at naming transformed food with respect to non-living things in bvFTD patients (p = .04, Z = -2.3), and no other significant difference was observed. Below we report descriptive statistics divided by patients group.*

| ***Word-picture matching*** | |  |  |  |  |
| --- | --- | --- | --- | --- | --- |
|  |  |  |  |  |  |
|  |  |  |  |  |  |
| ***bvFTD*** |  |  |  |  |  |
|  | **N** | **Min** | **Max** | **Mean** | **Std. Dev.** |
| Food | 10 | 33.00 | 36.00 | 34.90 | 1.29 |
| Nonfood | 10 | 15.00 | 36.00 | 32.80 | 6.32 |
| Natural food | 10 | 16.00 | 18.00 | 17.40 | 0.84 |
| Transformed food | 10 | 17.00 | 18.00 | 17.50 | 0.53 |
| Living | 10 | 0.00 | 18.00 | 16.10 | 5.67 |
| Nonliving | 10 | 15.00 | 18.00 | 16.70 | 1.06 |
|  |  |  |  |  |  |
| ***AD*** |  |  |  |  |  |
|  | **N** | **Min** | **Max** | **Mean** | **Std. Dev.** |
| Food | 11 | 34.00 | 36.00 | 35.55 | 0.69 |
| Nonfood | 11 | 33.00 | 36.00 | 35.00 | 1.18 |
| Natural food | 11 | 17.00 | 18.00 | 17.82 | 0.40 |
| Transformed food | 11 | 16.00 | 18.00 | 17.73 | 0.65 |
| Living | 11 | 16.00 | 18.00 | 17.55 | 0.69 |
| Nonliving | 11 | 16.00 | 18.00 | 17.45 | 0.82 |
|  |  |  |  |  |  |
| ***PPA*** |  |  |  |  |  |
|  | **N** | **Min** | **Max** | **Mean** | **Std. Dev.** |
| Food | 11 | 23.00 | 36.00 | 31.45 | 4.23 |
| Nonfood | 11 | 22.00 | 36.00 | 31.73 | 4.31 |
| Natural food | 11 | 9.00 | 18.00 | 16.00 | 2.57 |
| Transformed food | 11 | 9.00 | 18.00 | 15.45 | 2.62 |
| Living | 11 | 11.00 | 18.00 | 16.00 | 2.28 |
| Nonliving | 11 | 11.00 | 18.00 | 15.73 | 2.20 |

***S5.* Behavioural analysis on Categorization task**

*a) We ran a Mann-Whitney U Test with patients divided in bvFTD, PPA and Alzheimer’s disease, that is shortened as AD, and healthy controls, shortened as HC. No significant difference was observed in any of the patients’ groups performance. When compared to healthy controls, bvFTD patients performed worse at trials requiring to categorize transformed food and nonliving things as belonging to different categories (p = .04, U = 65), at trials requiring to categorize natural foods (p =.01, U = 52) as ‘same’, and at trials requiring to categorize living things as ‘same’ (p = .02, U = 55.5). Alzherimer’s patients’ accuracy did not differ from healthy controls’. PPA patients’ accuracy was lower for trials requiring to categorize transformed food and living things as different (p = .04, U = 48), at trials requiring to categorize living as ‘same’ (p = .002, U = 29), and showed a trend towards impairment at trials requiring to categorize living things and natural food as different (p = .056, U = 50).*

*b) Wilcoxon rank-sum test revealed that Alzheimer’s disease patients had greater difficulties at trials requiring to categorize natural food and living things as different, if compared to trials requiring to categorize natural food and nonliving things as different (p = .03, Z = -2.4). PPA patient’s performance was worse at correctly categorizing nonliving things versus living things (p = .02, U =* -2.5*). bvFTD showed no differences across categories.*

*Below we report descriptive statistics for each of the groups. It is to note, as the descriptive statistics table depicts, that all patients’ groups, even when performing significantly worse than healthy controls, did not show a great difference in the scores. Indeed, this task is less difficult than the naming task.*

| ***Categorization*** |  |  |  |  |  |
| --- | --- | --- | --- | --- | --- |
|  |  |  |  |  |  |
| ***bvFTD*** |  |  |  |  |  |
|  | **N** | **Min** | **Max** | **Mean** | **Std. Dev.** |
| Naltural food - Nonliving | 10 | 15.00 | 18.00 | 17.40 | 1.07 |
| Transformed food - Nonliving | 10 | 15.00 | 18.00 | 17.20 | 1.03 |
| Natural food - Living | 10 | 13.00 | 18.00 | 16.90 | 1.66 |
| Transformed food- living | 10 | 10.00 | 18.00 | 16.70 | 2.54 |
| Natural food | 10 | 14.00 | 18.00 | 16.70 | 1.42 |
| Transformed food | 10 | 15.00 | 18.00 | 17.70 | 0.95 |
| Living | 10 | 5.00 | 18.00 | 10.90 | 5.24 |
| Nonliving | 10 | 9.00 | 18.00 | 15.50 | 3.57 |
|  |  |  |  |  |  |
| ***AD*** |  |  |  |  |  |
|  | **N** | **Min** | **Max** | **Mean** | **Std. Dev.** |
| Naltural food - Nonliving | 11 | 18.00 | 18.00 | 18.00 | 0.00 |
| Transformed food - Nonliving | 11 | 16.00 | 18.00 | 17.73 | 0.65 |
| Natural food - Living | 11 | 14.00 | 18.00 | 16.55 | 1.57 |
| Transformed food- living | 11 | 17.00 | 18.00 | 17.64 | 0.50 |
| Natural food | 11 | 15.00 | 18.00 | 17.64 | 0.92 |
| Transformed food | 11 | 17.00 | 18.00 | 17.82 | 0.40 |
| Living | 11 | 6.00 | 18.00 | 14.00 | 4.94 |
| Nonliving | 11 | 6.00 | 18.00 | 14.91 | 4.30 |
|  |  |  |  |  |  |
| ***PPA*** |  |  |  |  |  |
|  | **N** | **Min** | **Max** | **Mean** | **Std. Dev.** |
| Naltural food - Nonliving | 8 | 13.00 | 18.00 | 17.00 | 1.77 |
| Transformed food - Nonliving | 8 | 16.00 | 18.00 | 17.50 | 0.76 |
| Natural food - Living | 8 | 15.00 | 18.00 | 16.63 | 1.19 |
| Transformed food- living | 8 | 16.00 | 18.00 | 17.13 | 0.83 |
| Natural food | 8 | 16.00 | 18.00 | 17.38 | 0.74 |
| Transformed food | 8 | 17.00 | 18.00 | 17.63 | 0.52 |
| Living | 8 | 8.00 | 17.00 | 13.63 | 3.42 |
| Nonliving | 8 | 14.00 | 18.00 | 16.75 | 1.49 |

***S6.*** *Sensory-functional matching task: behavioural data of a repeated measures ANOVA with patients divided in bvFTD, PPA and Alzheimer’s disease, shortened as AD, and healthy controls (HC).*

The repeated measures ANOVA revealed *first*, a significant main effect of *Group* [*F*_(3, 54)_ = 38.31, *p* < .001, η2 = .31], with healthy controls outperforming bvFTD (*p* =.001, .29 .1.58, 95% of CI), Alzheimer’s disease (*p* = .003, .22, 1.47, 95% of CI) and PPA (*p* =.05, 0. 1.25, 95% of CI). We found a main effect of *type of food* [*F*_(3, 54)_ = 8.35, *p* = .006, η2 = .13], with transformed food trials showing a better performance than natural food trials (.1, .52, 95% of CI). We also found a significant interaction *type of food * property* [*F*_(3, 54)_ = 7.37, *p* = .009, η2 = .12] with a better performance at natural food in the sensory block if compared to the functional block (*p* = .01, with .09, .68 95% of CI). In addition, a significant three-way interaction emerged between *type of food * property * group* [*F*_(3, 54)_ = 5.60, *p* = .002, η2 = .24], with bvFTD and PPA performing better at natural food trials in the sensory block (congruent), with respect to natural food trials in the functional block (incongruent), [( *p* = .002 , .44, 1.76 95% of CI) and (*p* = .048, .01, 1.27, 95% of CI) respectively]. Conversely, Alzheimer’s disease patients do not show differences between congruent and incongruent trials for either natural, either transformed food, while controls perform almost at ceiling, not showing any difference.

*Below we report descriptive statistics for each of the groups.*

| ***Sensory-functional matching*** |  |  |  |  |  |
| --- | --- | --- | --- | --- | --- |
|  |  |  |  |  |  |
| ***bvFTD*** |  |  |  |  |  |
|  | **N** | **Min** | **Max** | **Mean** | **Std. Dev.** |
| Sensory- natural food | 10 | 5.00 | 7.00 | 6.20 | 0.92 |
| Sensory - transformed food | 10 | 3.00 | 7.00 | 5.80 | 1.32 |
| Functional - Natural food | 10 | 3.00 | 7.00 | 5.10 | 1.60 |
| Functional - Transformed food | 10 | 6.00 | 7.00 | 6.20 | 0.42 |
|  |  |  |  |  |  |
| ***AD*** |  |  |  |  |  |
|  | **N** | **Min** | **Max** | **Mean** | **Std. Dev.** |
| Sensory- natural food | 11 | 3.00 | 7.00 | 5.73 | 1.27 |
| Sensory - transformed food | 11 | 5.00 | 7.00 | 6.09 | 0.83 |
| Functional - Natural food | 11 | 3.00 | 7.00 | 5.73 | 1.35 |
| Functional - Transformed food | 11 | 5.00 | 7.00 | 6.09 | 0.70 |
|  |  |  |  |  |  |
| ***PPA*** |  |  |  |  |  |
|  | **N** | **Min** | **Max** | **Mean** | **Std. Dev.** |
| Sensory- natural food | 11 | 4.00 | 7.00 | 6.18 | 1.25 |
| Sensory - transformed food | 11 | 4.00 | 7.00 | 6.27 | 1.19 |
| Functional - Natural food | 11 | 3.00 | 7.00 | 5.55 | 1.29 |
| Functional - Transformed food | 11 | 5.00 | 7.00 | 6.55 | 0.69 |

***S7. Naming: a) nonliving things VBM results.*** K = cluster size (number of voxels).

***
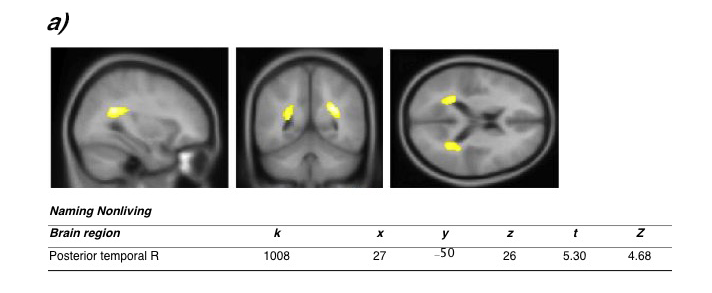
***

***b) TBSS conjunction analysis results: the crosshair corresponds to the peak coordinates of the significant cluster.***

***
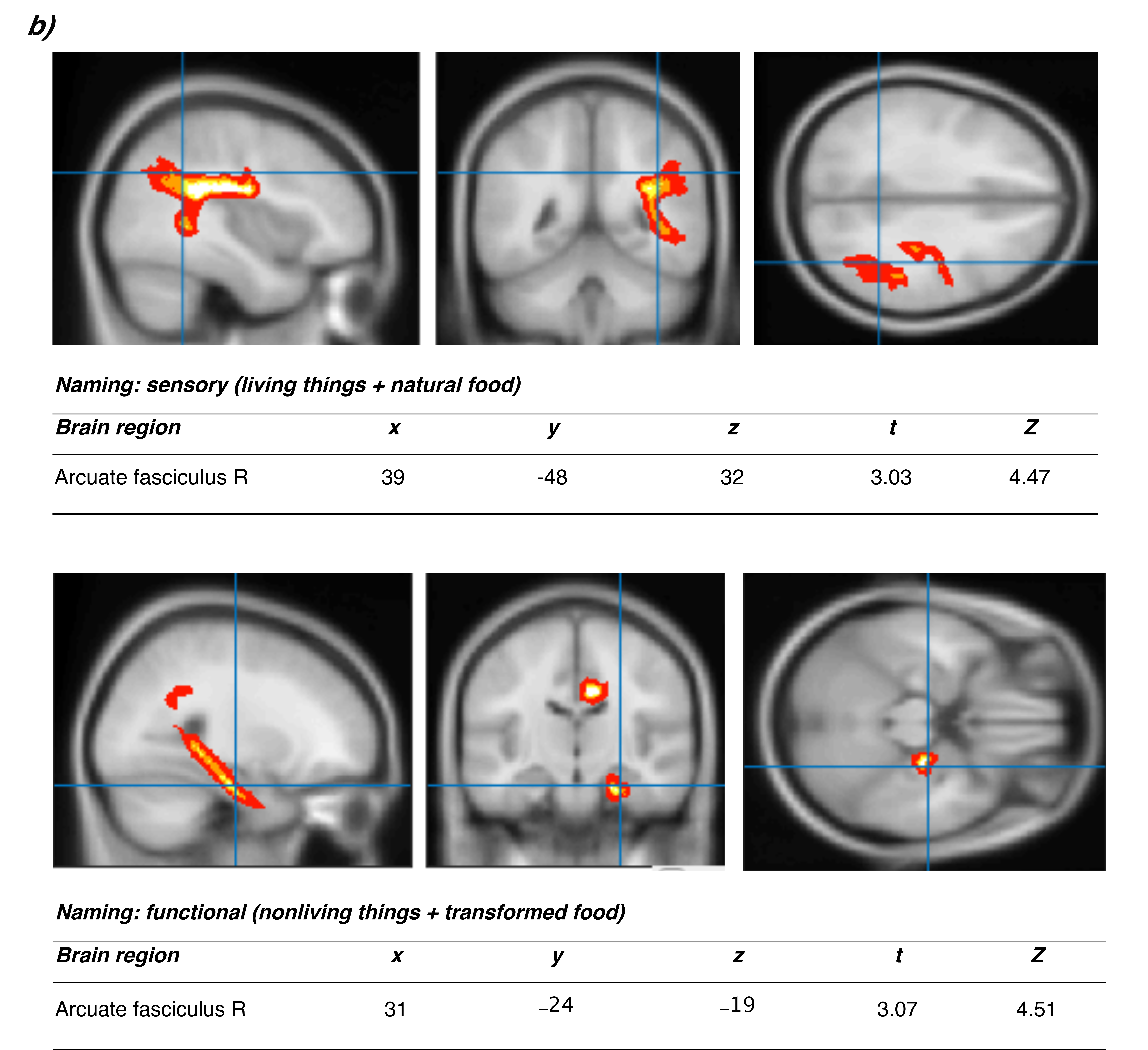
***

*In addition, DTI tractography analyses supported these results, with a positive correlation between the volume of the right arcuate fasciculus and naming ‘sensory’ items (r=.45, p =.001) and the right cingulum’s volume and naming ‘functional’ items (r =.3, p=.05). See also table in S12.*

***S8. VBM and TBSS results for the sensory-functional matching task. a, b show results for the functional block. c shows TBSS results only for the sensory block. For the TBSS, the crosshair corresponds to the peak coordinates of the significant cluster.*** K = cluster size (number of voxels).

***

***

*DTI analyses confirm a positive correlation between the volume of the left IFOF and accuracy at matching transformed foods with functional words (r=.34, p= .02). See also S12.*

***S9.*** *Correlation coefficients for GM mean values extracted from the VBM analyses’ results and the behavioural variables of interest. It is interesting to note that not only the mean volume at the significant cluster (computed for each subject) significantly correlates with its behavioural variable of interest, but also with related variables of interest: in fact, the volume for the word-picture matching “functional’ analysis, correlates with word-picture matching ‘functional’ performance, but also with performance on transformed food trials and on functional-transformed food trials of the sensory-functional matching task. (This correlation matrix does not include naming and categorization task because some of our participants did not perform those tasks). (wpic.F = word picture matching functional; wpic.NF = word-picture matching natural food; wpic.TF = = word-picture matching transformed food).*

|  | | | | | | | | |  |
| --- | --- | --- | --- | --- | --- | --- | --- | --- | --- |
|  | **GM.wpic.F** | **wpic.F** | **GM.wpic.NF** | **wpic.NF** | **GM.wpic.TF** | **wpic.TF** | **GM.sfF** | **sfF** |  |
| **GM.wpic.F** | 1.000 | .349^**^ | -0.150 | 0.214 | .649^**^ | .360^**^ | .473^**^ | 0.110 |  |
| **wpic.F** | .349^**^ | 1.000 | -0.196 | 0.248 | 0.049 | .463^**^ | 0.180 | 0.142 |  |
| **GM.wpic.NF** | -0.150 | -0.196 | 1.000 | .408^**^ | 0.250 | -0.057 | 0.103 | 0.040 |  |
| **wpic.NF** | 0.214 | 0.248 | .408^**^ | 1.000 | 0.099 | .712^**^ | .281^*^ | 0.239 |  |
| **GM.wpic.TF** | .649^**^ | 0.049 | 0.250 | 0.099 | 1.000 | 0.108 | .443^**^ | 0.083 |  |
| **wpic.TF** | .360^**^ | .463^**^ | -0.057 | .712^**^ | 0.108 | 1.000 | .275^*^ | 0.195 |  |
| **GM.sfF** | .473^**^ | 0.180 | 0.103 | .281^*^ | .443^**^ | .275^*^ | 1.000 | .346^**^ |  |
| **sfF** | 0.110 | 0.142 | 0.040 | 0.239 | 0.083 | 0.195 | .346^**^ | 1.000 |  |
| **. Correlation is significant at the 0.01 level (2-tailed). | | | | | | | | | |
| *. Correlation is significant at the 0.05 level (2-tailed). | | | | | | | | | |

*B) Also categorization ‘sensory’ items correlated with GM concentration at the cluster of interest (r =.27, p =.02).*

***S10.*** *Results from the same VBM analysis models described in the main text, but performed in each of the three patients groups, separately, compared to controls. In the figure K = cluster size (number of voxels).*

1. ***bvFTD patients.*** ***Naming task.*** *The VBM analysis highlighted, in the comparison between bvFTD patiens and healthy controls, a significant cluster correlating with nonliving things naming peaking in the posterior right cingulum. No other significant correlations emerged at this task.* ***Word-picture matching task****: a significant cluster emerged for ‘functional knowledge’ at the conjunction analysis (nonliving + transformed food) in the bilateral parahippocampal gyrus, consistent with the behavioural impairment with respect to controls, at nonliving things and transformed foods in this task.* ***Categorization task****: consistently with the two previous results, a significant cluster emerged for ‘functional knowledge’, peaking in the left cerebellum and extending to the posterior temporal region, and for transformed food, peaking in the left cerebellum and extending to the left fusiform gyrus, consistent with the impaired performance at trials with transformed food and nonliving things, among the others, and with palatable foods recognition. See the figure below for details, coordinates and cluster size.*

*
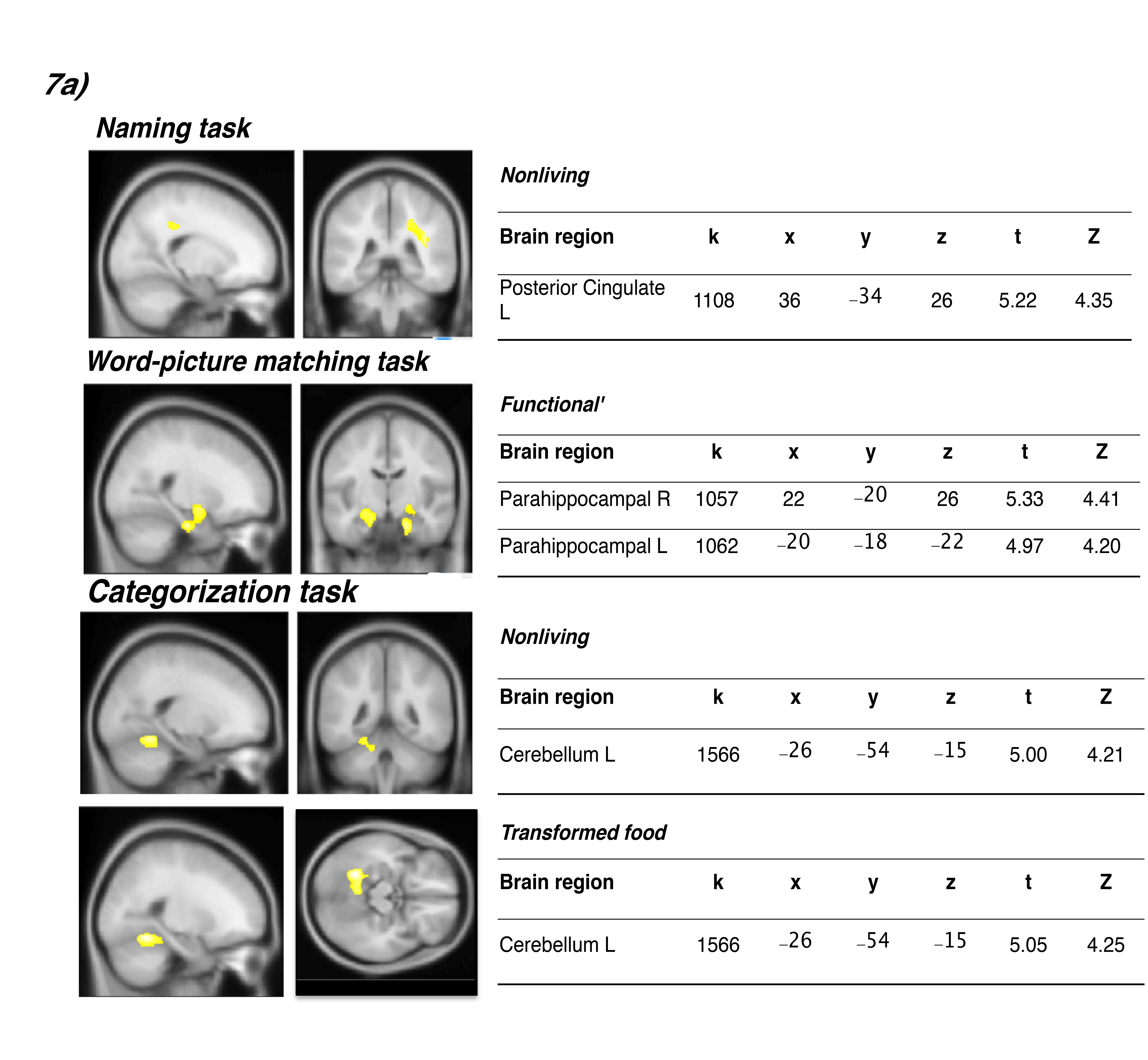
*

1. ***Alzheimer’s disease patients.*** *Naming task: a significant cluster emerged peaking in the right putamen, when correlating local grey matter concentration with living things naming perfomance compared to healthy controls, and consistent with the overall behavioural impairment of patients with living things vs. nonliving things (see S3 for the repeated measures ANOVA between the four groups).* ***Word-picture matching task****: we found significant clusters correlating with recognition of nonliving things peaking in the right rectum and extending to the frontal cortex bilaterally, consistently with the literature on tool use (e.g. 13, 14) and with their worse performance with non-food in general; we also found a significant cluster correlating with food recognition (left LOC), and one with ‘functional knowledge’ (peaking in the left occipital cortex and extending to the pMTG).* ***Categorization task****: only a large cluster emerged for ‘sensory knowledge’, in the right lateral occipital cortex (see table below for coordinates and details), peaking in the left calcarine fissure.*

*
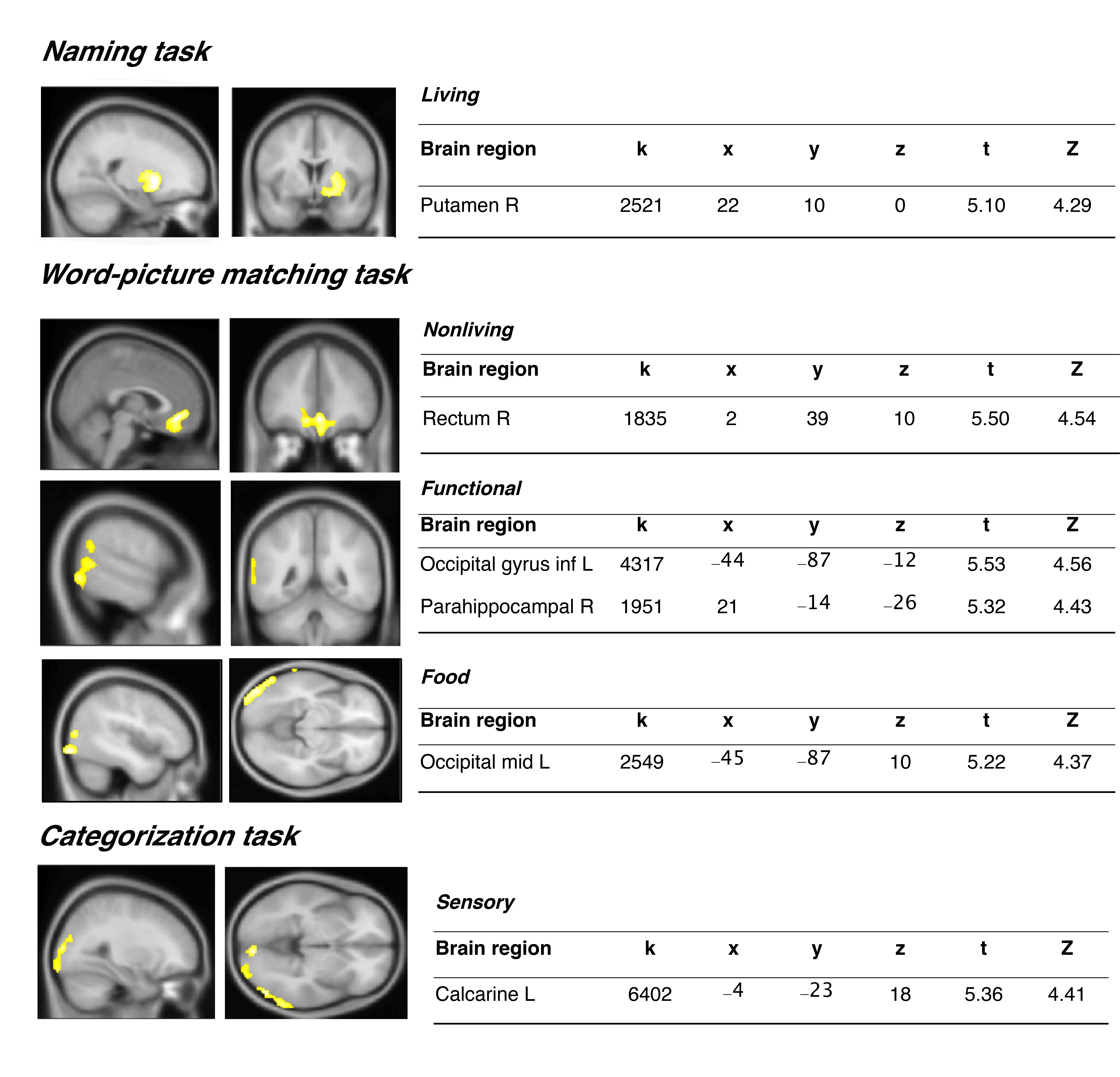
*

1. ***Primary progressive aphasia patients*.** ***Naming task****: significant clusters emerged for natural food naming (k > 1000) and living (> 600), with peaks in the orbitofrontal cortex and in the mid LOC, respectively, consistently with our hypotheses and with what proposed in the literature about living things recognition.* ***Word-picture matching task:*** *behaviourally, PPA patients were more impaired with respect to controls in all categories. The VBM highlighted* *a large cluster peaking in the lateral occipital cortex (right) when correlating recognition accuracy for natural foody with local GM concentration in comparison with healthy controls. For transformed food, we observed a significant cluster in the bilateral caudate, previously associated to palatable food viewing. A large cluster in the left insula and subgyral white matter emerged for living things recognition, and, peaking in the ACC and in the occipital cortex, we found a cluster for ‘sensory knowledge’ . In addition, accurate food recognition correlates with local GM contration in the LOC bilaterally, right precuneus, bilateral angular gyrus, consistently with the literature about food recognition (24). Categorization task: nothing survived FWE correction.*

*
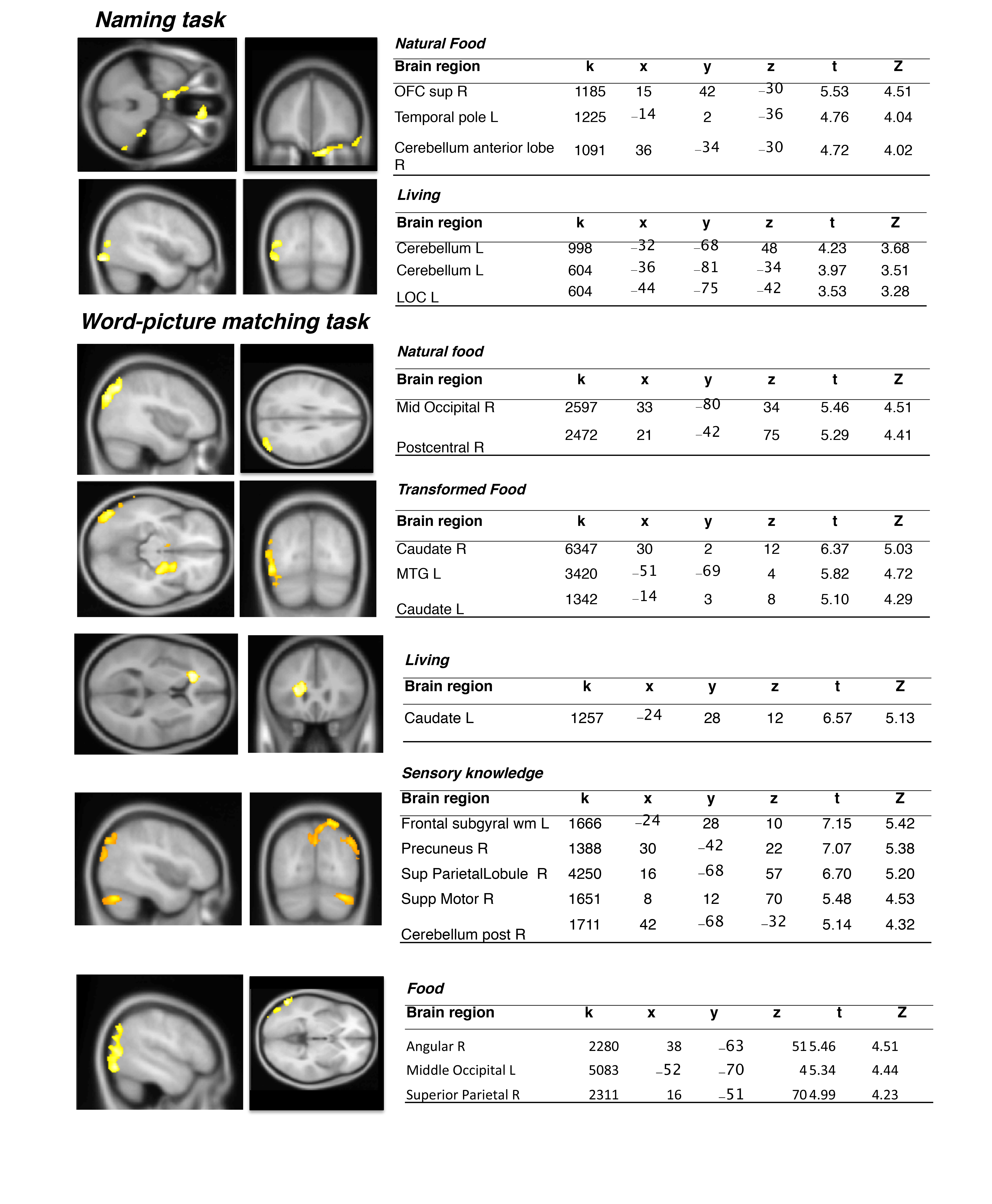
*

***S11.*** *Results from the VBM analysis performed in the three patients groups separately, compared to controls. The model applied was, in this case, an ANOVA, in order to highlight overall grey matter differences for each group. It is to note that the regions found involved for each patients’ group, are consistent with those emerged from the analysis reported in s10 and with the regions reported across the main text for the categories of interest. Peak coordinates are reported. In the figure K = cluster size (number of voxels). a) PPA patients disclose an extensive temporal atrophy peaking in the left superior temporal gyrus and extending to the temporal lobe (bilaterally) and to the left inferior frontal gyrus, consistently with this type of diagnosis. b) AD patients disclose and extensive hippocampal bilateral atrophy, extending to temporal regions, and posterior parietal atrophy, with its peak in the precuneus. c) bvFTD patients differ significantly from controls only in the left STG and hippocampus, and in the cerebellum. The overall pattern of atrophy of this group was less marked with respect to PPA and AD, as one could expect based on their less pronounced impairment at some of the tasks.*

*
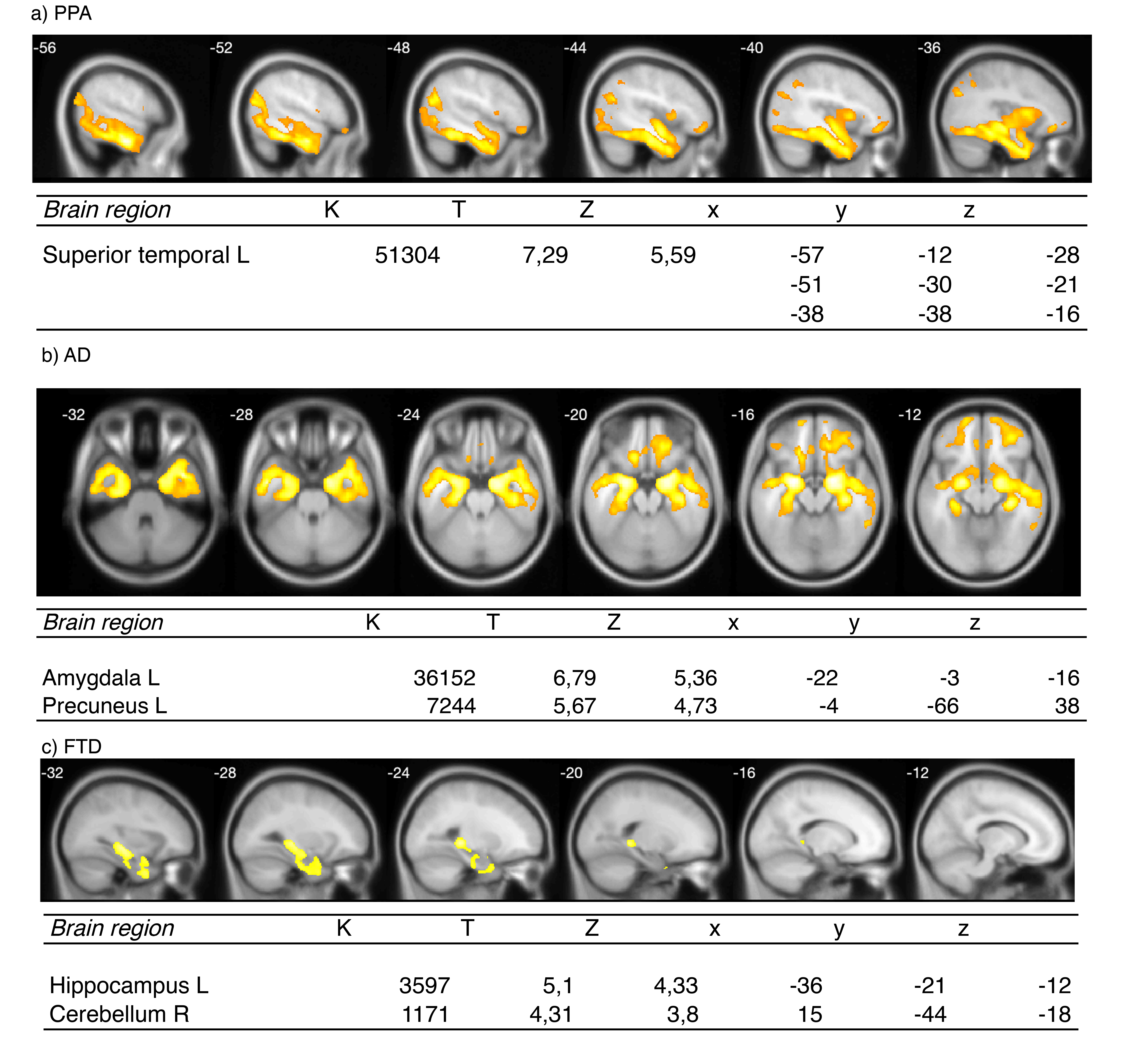
*

***S12.*** *Correlation and regression analyses conducted between behavioural variables of interest and volume of the white matter tracts (DTI tractography).*

|  |  |  |  |  |  |  |
| --- | --- | --- | --- | --- | --- | --- |
|  |  |  |  |  |  |  |
| ***Regression Models*** |  |  |  |  |  |  |
|  |  |  |  |  |  |  |
|  |  |  |  |  |  |  |
|  |  |  |  |  |  |  |
| **Dependent variable** | **Predictors** | **R2** | **Beta** | **t** | **Sig.** |  |
| *Categorization: sensory* |  | .239 |  | 4.476 | <.001 |  |
|  |  |  |  |  |  |  |
|  | Left cingulum |  | 0.15 | 0.831 | 0.411 |  |
|  | Right cingulum |  | -0.402 | -2.263 | 0.029 |  |
|  | Corpus Callosum |  | 0.425 | 2.881 | 0.006 |  |
|  | Right IFOF |  | -0.015 | -0.103 | 0.918 |  |
|  |  |  |  |  |  |  |
|  |  |  |  |  |  |  |
| **Dependent variable** | **Predictors** | **R2** | **Beta** | **t** | **Sig.** |  |
| *Word-picture: Functional* |  | 0.32 |  | 31.03 | <.001 |  |
|  |  |  |  |  |  |  |
|  | Right cingulum |  | 0.46 |  | 0.001 |  |
|  | Right IFOF |  | -0.29 |  | 0.025 |  |
|  |  |  |  |  |  |  |
| **Dependent variable** | **Predictor** | **R2** | **Beta** | **t** | **Sig.** |  |
| *SF matching: Functional-Transfomed* |  | 0.1 |  | 68.56 | <.001 |  |
|  |  |  |  |  |  |  |
|  | Left IFOF |  | -0.32 |  | 0.03 |  |
|  |  |  |  |  |  |  |
|  |  |  |  |  |  |  |
| ***Correlations (Spearman's rho)*** |  |  |  |  |  |  |
|  |  |  |  |  |  |  |
|  |  |  |  |  |  |  |
| **Naming Functional** | **R cingulum** |  |  |  |  |  |
| *r* | 0.28 |  |  |  |  |  |
| *p* | 0.05 |  |  |  |  |  |
|  |  |  |  |  |  |  |
| **Naming Sensory** | R arcuate |  |  |  |  |  |
| *r* | 0.37 |  |  |  |  |  |
| *p* | 0.01 |  |  |  |  |  |
